# Reduced‐Graphene Oxide Nanofiltration Membranes Intercalated by Conjugated Polyaromatics: Towards High Monovalent Salt Rejections

**DOI:** 10.1002/advs.202523231

**Published:** 2026-03-26

**Authors:** Muskan Sonker, Sankar Nair

**Affiliations:** ^1^ School of Chemical & Biomolecular Engineering Georgia Institute of Technology Atlanta Georgia USA

**Keywords:** graphene oxide, intercalation, membranes, nanofiltration, salt rejection

## Abstract

Graphene oxide (GO) nanofiltration (NF) membranes have emerged as promising candidates for processing of aqueous streams, due to fast water transport characteristics and excellent chemical stability in harsh operating environments. Their practical applications under realistic high salt/solute concentration conditions are challenged by a lack of systematic approaches to control selectivity, as well as membrane swelling and mechanical stability. Here, we show that intercalation of rGO (reduced GO) membranes with polyaromatic conjugated molecules of different structural classes is an effective strategy to enhance and tune their performance. Specifically, these membranes allow entry into a “deep NF” regime with remarkably high inorganic monovalent salt rejections (NaCl, 75–85%) and near‐total divalent salt rejections (Na_2_SO_4_, ∼98%) in a large concentration range (0.01–0.1 M) while maintaining high water permeability.

## Graphical abstract

We demonstrate the fabrication of a series of reduced‐GO nanofiltration membranes containing intercalated polyaromatic conjugated molecules of different structural classes. These tuned‐nanochannel membranes perform “deep nanofiltration” with remarkably high monovalent salt rejections and near‐total divalent salt rejections in a large concentration range while maintaining high water permeability. They display excellent robustness and performance in real petroleum refinery wastewater streams.

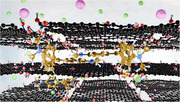

## 1 Introduction

Nanofiltration (NF) membranes are widely used in desalination processes, with their molecular weight cut‐offs (MWCOs) [1, 2] ranging between 200–1000 Daltons. A key functional difference between NF and reverse osmosis (RO) membranes (MWCOs < 200 Da) [3, 4, 5] lies in their selectivity: while NF membranes primarily reject multivalent ions and organic solutes, RO membranes can additionally remove small monovalent salts such as NaCl. Tuning of NF membranes to access lower MWCOs, *i.e*., “deep NF”, could significantly benefit both industrial water processing and desalination. Many industrial processes require sub‐potable water quality, while in desalination, deep NF can debottleneck downstream RO units [4, 6]. Since NF membranes operate at substantially lower pressures than RO, this strategy could deliver notable energy and cost savings. At the same time, conventional polymeric NF membranes are increasingly challenged in complex feed streams containing high dissolved solids, and at higher industrial process temperatures and pH extremes [7, 8, 9]. Among non‐polymeric NF membranes, graphene oxide (GO)‐based membranes have emerged as a promising technology due to their intrinsically high chemical and temperature resistance, and tunable properties [10, 11, 12].

A key route for tuning GO and reduced‐GO (rGO) membrane microstructure is the intercalation of guest species in the interlayer spaces between the GO nanosheets [13, 14, 15, 16]. Several techniques are known for intercalating GO membranes, including in situ intercalation, suspension mixing, and layer‐by‐layer assembly [17, 18, 19, 20, 21, 22]. A wide range of materials have been intercalated, such as organic molecules [23, 24, 25, 26], polymers [27, 28, 29, 30], inorganic nanoparticles [31, 32], ions [24, 33, 34, 35], and 2D materials [36, 37, 38, 39]. These intercalants can control the interlayer spacing, modify the pore size distribution, and introduce additional chemical functionalities. However, a systematic understanding of relationships between intercalant composition, membrane microstructure, and separation properties remains a challenge [40, 41, 42, 43, 44, 45]. Our work on intercalated GO membranes focuses on well‐defined polyaromatic conjugated (“PAC”) molecules [45, 46, 47, 48], which also constitute a large group of industrially available dyes/colorants. PACs strongly bind to a variety of surfaces through π‐π, π‐cation, and direct coulombic interactions [49], and exhibit strong bonding on GO/rGO surfaces. PAC intercalants can allow many GO membrane microstructures and functionalities to be systematically constructed and studied. We recently showed [47, 50] that 7‐amino‐8‐methylphenothiazin‐3‐ylidene)‐dimethylazanium chloride, a thiazine‐derived PAC also known as toluidine blue O (TBO), forms multiple GO/rGO membrane microstructures due to a variety of interlayer TBO arrangements. This led to non‐intuitive and favorable flux and solute rejection behavior. These membranes were scaled to large sheets and operated reliably in real feed streams for hundreds of hours [44, 50]. More recently, a combination of UV‐vis spectroscopy, fluorescence spectroscopy, x‐ray diffraction, and permeation measurements revealed a wide variety of specific TBO arrangements, which could be controlled by the GO/rGO:TBO ratio in the suspension/ink used for membrane deposition [46].

**SCHEME 1:**
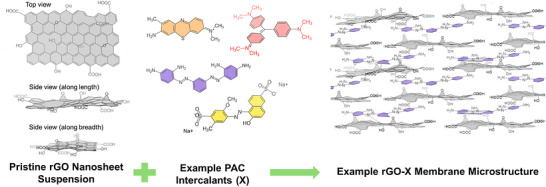
Schematic view (not‐to‐scale) of polyaromatic conjugated molecule (denoted X) intercalation to produce rGO‐X membranes.

In the present study, we explore the effects of PAC type on the GO/rGO membrane microstructure and separation properties. The large number of available PAC molecules [49] can be categorized into a few molecular classes. Three important classes are thiazines, azos, and triarylmethanes (all illustrated in Scheme 1 and Table 1). Our hypothesis is that the different structures of these three classes of molecules would have significantly different effects on the interlayer spaces due to differences in binding environments, packing, and aggregation (e.g., dimers); thereby allowing variation of water and solute transport. We have selected four intercalants (Table 1) labeled AZ1, AZ2 (both azo class), TM1 (triarylmethane class), and TZ1 (TBO, as a benchmark representative of the thiazine class). We fabricate GO‐X and rGO‐X membranes (X = intercalant) on porous poly(ethersulfone) (PES) substrates. To enable valid comparisons, we fabricate all membranes with the same molar content of the intercalant (i.e., number of intercalated molecules per unit mass of GO/rGO). We systematically characterize their microstructure and permeation/nanofiltration properties, and explain the key trends and tunability of these properties in terms of the microstructure. Specific membranes show highly attractive salt rejection characteristics over and above the state of the art, including our prior findings with rGO‐TZ1 [45, 50] membranes.

**TABLE 1:** Structural and molecular characteristics of the intercalants used in this study, including short label, class, common name, IUPAC name, molecular weight, polyconjugated network size, dimensions, effective surface area, and structure.

Intercalant Label, Class, Common Name	IUPAC Name	MW (g/mol) # of conjugated atoms Dimensions (L × W × H), Å Effective 2D area (LxW), Å^2^	Structure
AZ1 Azo Bismarck Brown	4,4′‐[1,3‐phenylenebis(diazene‐2,1‐diyl)]di(benzene‐1,3‐diamine) dihydrochloride	419 26 18.2 × 8.8 × 2.6 160	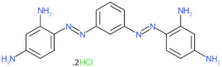
AZ2 Azo Allura Red AC	disodium 6‐hydroxy‐5‐((2‐methoxy‐5‐methyl‐4‐sulfophenyl)azo)‐2‐naphthalenesulfonate	496 20 14.7 × 8.0 × 3.7 118	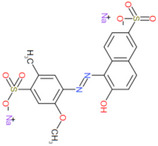
TM1 Triaryl‐methane Crystal Violet	Tris(4‐dimethylamino) phenyl) methylium chloride	408 22 14.5 × 13.8 × 3.9 200	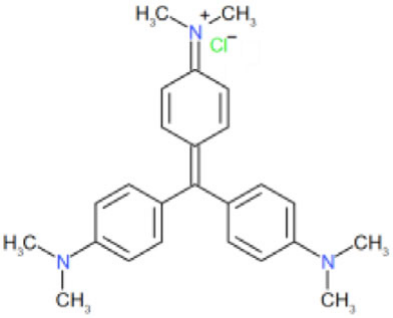
TZ1 Thiazine Toluidine Blue O	(7‐amino‐8‐methylphenothiazin‐3‐ylidene)‐dimethylazanium chloride	306 16 13.7 × 5.5 × 2.8 75	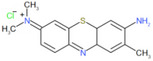

## 2 Results and Discussion

Table 1 shows the intercalant molecular structure, molecular mass, molecular class, names (label used in this work, IUPAC name, and commonly used name); and other characteristics such as molecular dimensions, number of π‐conjugated atoms, and effective 2D area. The dimensions were calculated by summing the distances between the centers of the farthest atoms in each direction (*x*, *y*, and *z*) and incrementing these distances with the relevant atomic radii. Molview renderings of each intercalant were generated to visualize their 3D structure (Figure S1a–d). Among the intercalants, AZ1 exhibits the highest planarity with the smallest *z*‐dimension of 2.6 Å, which is due to sp^2^ hybridization of its terminal ─NH_2_ groups, which remain coplanar with the aromatic backbone. TZ1 (2.8 Å) and AZ2 (3.7 Å) also maintain largely planar structures, with only modest deviations due to their substituents. In contrast, TM1 has a bulky triarylmethane framework and adopts a propeller‐like [51, 52, 53, 54] configuration in vacuum (Figure S1d) due to steric hindrance between ortho‐protons on the phenyl rings. Prior density functional theory calculations on TM1 [55] confirm its greater flexibility relative to the other intercalants. Table 1 provides an initial structural basis for discussing their potential influence on the rGO interlayer spaces. The z‐dimension offers a qualitative measure of out‐of‐plane thickness that may contribute to differences in gallery spacing; whereas the lateral dimensions and conjugated network size influence the extent of π–π binding with GO/rGO, which in turn affects packing efficiency and the ability to suppress or promote interlayer swelling. It should be noted that these dimensions are derived from isolated molecular structures and do not fully capture the complexity of intercalant behavior in the interlayer spaces, which moreover, will include the presence of water.

Figure S2 shows photographs of equilibrated aqueous solutions of the intercalants (X = TZ1, TM1, AZ1, AZ2) at a range of concentrations, as well as corresponding equilibrated rGO‐X suspensions after addition of a fixed amount of rGO. Figures S3 and S4 show the UV–vis absorption spectra of all these solutions and suspensions. We deconvoluted all the intercalant solution spectra using Gaussian peak fitting and plotted the integrated areas of each peak as a function of intercalant concentration (Figures S5–S8). As described in detail in our recent work [46] and previous studies [50], TZ1 shows a variety of peaks as a function of concentration (Figure S5). These include a single absorption peak of the monomeric form, red‐shifted peaks belonging to laterally interacting TZ1 molecules (known as J‐aggregates), and blue‐shifted peaks belonging to vertically stacked TZ1 molecules (H‐aggregates). The proportion of these forms changes non‐linearly with concentration, with H‐ and J‐aggregates dominating at higher concentrations. The absorption spectrum of TM1 (Figure S6) shows two distinct peaks at ∼590 and ∼540 nm, which are consistent with literature assignments to the monomeric species and the H‐aggregate, respectively [56]. The proposed TM1 dimer arrangement [56] is shown in Figure S6k. It maximizes separation of positive charges, leading to stacking of terminal substituents but not the entire molecules (i.e., a “Z‐shaped” arrangement). On the other hand, AZ1 and AZ2 do not form dimeric aggregates (Figures S7 and S8). The observed peaks are attributed to electronic absorptions of the monomer, consistent with prior studies [57, 58, 59].

Figures S9 and S10 show fluorescence emission spectra with the four equilibrated intercalant solutions at different concentrations, as well as the corresponding equilibrated rGO‐X suspensions. In the case of suspensions, the plots indicate the overall intercalant concentration in the suspension (rGO‐surface‐adsorbed intercalant and solution‐phase intercalant in equilibrium with the bound phase). Figure S11 shows the fluorescence emission area intensities for each intercalant solution and the corresponding rGO‐X suspension. Due to the binding of the intercalants on the rGO nanosheet surfaces, the fluorescence is quenched. For each value of total intercalant concentration, the difference between the fluorescence intensity of the intercalant solution and the rGO‐X suspension gives the fraction of the intercalant molecules adsorbed on rGO (i.e., quenched), and the remainder consists of dissolved intercalant in equilibrium with the adsorbed phase. These results are depicted as isotherms in Figure S12. In the concentration ranges used here, we find that TM1, TZ1, and AZ1 adsorb with high capacity (Q_eq_) on rGO, with negligible solution‐phase concentrations (*C*_eq_) at equilibrium. However, AZ2 shows a lower adsorption strength on rGO. We attribute this to the negatively charged sulfonate groups and the steric hindrance of these bulky, non‐planar groups.

**FIGURE 1:**
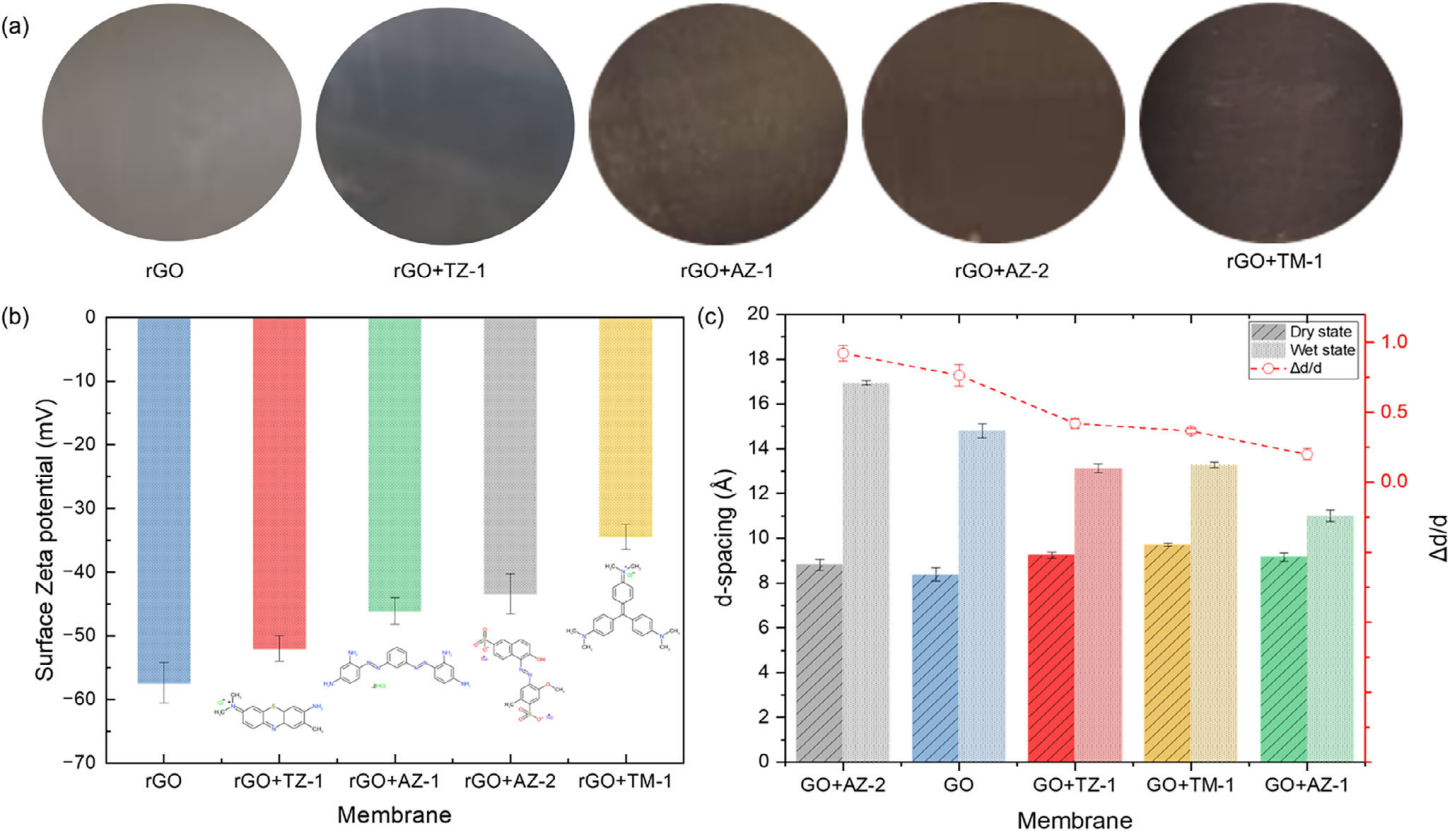
(a) Visuals of rGO and rGO‐X (X: intercalant/molecule) membranes used in this work (coupon diameter is 47 mm). (b) Surface zeta potential of intercalated rGO membranes at neutral pH and 25°C. (c) Evolution of GO and GO‐X membranes interlayer d‐spacing for compacted membranes under dry and wet states.

Four types of rGO‐X membranes were fabricated on macroporous poly(ethersulfone) (PES, 30 nm nominal pore size) supports using the same molar amounts of intercalant per unit mass of rGO in the suspension, specifically 0.33 mmol X/g rGO. This enables direct comparison of the membrane properties with the same number of intercalant molecules within the membranes. In terms of intercalant mass fraction in the membranes, this corresponds to 9.1 wt% (rGO‐TZ1), 11.8 wt% (rGO‐TM1), 12.1 wt% (rGO‐AZ1), and 14.0 wt% (rGO‐AZ2). Bare rGO membranes (no intercalant) were also fabricated. Figure S13 presents top‐view SEM images of the rGO‐X membranes on PES, demonstrating uniform and continuous coverage of the underlying support, as well as similar membrane thicknesses across the different rGO‐X membranes. Figure S14 displays cross‐sectional SEM images of the PES‐supported rGO‐X membranes, clearly revealing the distinct rGO‐X selective layer with an average thickness of 144±17 nm (rGO), 141± 19 nm (rGO‐TZ1), 140±21 nm (rGO‐TM1), 142±18 nm (rGO‐AZ1), and 145±22 nm (rGO‐AZ2). Photographs of the fabricated membranes are shown in Figure 1a. The solid‐state UV–vis absorbance spectra of the intercalated rGO‐X membranes (including deconvolution into individual peaks) are shown in Figure S15. These spectra demonstrate the different types of aggregates formed in the rGO environment. As shown in our earlier study [46], TZ1 (Figure S15a) exhibits signatures of both H and J‐type aggregates, due to its smaller conjugated network and z‐dimension that allows both lateral (J‐aggregate) stacking and partial face‐to‐face arrangements (H‐aggregate). In Figure S15b, TM1 also shows spectral signatures of both H‐ and J‐type aggregates, consistent with prior studies of TM1 aggregation [53]. On layered substrates, TM1 has been shown to form additional aggregate states, including H‐aggregates at ∼510 nm in zirconium phosphate [60], tightly packed H‐aggregates below 450 nm in clays, and J‐aggregates at 620–665 nm in polyphosphate matrices [58, 59, 60, 61]. In Langmuir–Blodgett films, both J‐dimers and H‐dimers as well as H‐tetramers have been resolved, confirming that multiple TM1 aggregate types coexist under 2D confinement [52]. In contrast, AZ1 (Figure S15c) displayed no aggregation signatures, consistent with prior studies showing that AZ1 primarily undergoes reversible trans‐cis isomerization rather than excitonic coupling [57]. Finally, AZ2 (Figure S15d) shows a broad band spanning 500–560 nm, most likely arising from microenvironmental broadening or adsorption‐induced shifts of the same chromophore, although weak lateral packing cannot be excluded.

Figure 1b shows the net surface zeta potentials of these intercalated membranes and the bare rGO membrane. All the intercalated membranes have a lower negative (i.e., more neutral) zeta potential relative to the bare rGO membrane (−57.4 ± 3.2 mV) at pH 7. Since the intercalants were incorporated at equal molar fractions, the observed differences in surface charge are attributable to the intrinsic acid‐base equilibria of the intercalant molecules and the manner in which their ionizable groups interact with the rGO surface and the surrounding interlayer environment. Intercalant TM1, with dimethylamino groups (pK_a_ 5.3, 8.6) [62], remains protonated below neutral pH, strongly cationic. It thus most effectively neutralizes surface ─COO^−^ charges (−34.4 ± 2 mV). TZ1 (pK_a_ 2.4, 11.6) [63] carries one strongly basic site (‐N(CH_3_)_2_) and one weakly basic site (phenothiazinium core); at pH 7, partial protonation gives weaker neutralization (−52 ± 2 mV). AZ1 (pK_a_ ∼4.7–5.0) is only moderately protonated at pH 7, yielding intermediate compensation (−46.1 ± 2 mV). In contrast, AZ2 contains two sulfonic acid groups (pK_a_ < 1, fully deprotonated and negatively charged at pH 7) and a phenolic ─OH with reported pK_a_ = 11.28 [64]. At pH 7, the phenol remains protonated while the sulfonates dominate the charge, making the dye strongly anionic. Nevertheless, AZ2 binds to rGO via π–π stacking, compressing the double layer and reducing the apparent zeta potential to −43.4 ± 3.2 mV.

Figure 1c shows the average interlayer *d*‐spacings (obtained by XRD measurements) of the corresponding GO‐based membranes after hydraulic conditioning, measured under both “dry” (oven dried at 35°C for 24 h) and “wet” (saturated with liquid water) conditions. The raw XRD spectra are shown in Figure S16. We use GO membranes as a proxy for rGO membranes for XRD measurements, since rGO membranes exhibit substantial disorder in nanosheet stacking/registry and return broad XRD reflections without distinct peaks. In the dry compacted state, the interlayer spacing is primarily determined by pressure‐assisted compaction, which dictates the extent to which the interlayer galleries can collapse in the absence of water (after drying). The pure GO membrane collapsed to the van der Waals limit, whereas the GO‐X membranes retained larger residual spacings due to pillaring by the guest molecules. In the wet compacted state, additional factors beyond pressure‐assisted compaction influence the spacing, including the hydration state of functional groups, steric contributions, and charge effects. Here, the observed order is GO‐AZ2 > GO > GO‐TZ1 ∼ GO‐TM1 > GO‐AZ1. Intercalant AZ2, containing deprotonated sulfonate groups (pKa < 1), showed the largest spacing due to strong hydration and steric contributions from its bulky substituents. TZ1, with a smaller footprint (∼75 Å^2^) and z‐dimension (∼2.8 Å), mainly formed monomers and lateral J‐aggregates that have attractive π–π interactions with the GO layers, thereby suppressing expansion in water. TM1 retained a wet spacing similar to TZ1. Its propeller configuration is expected to slightly expand the dry spacing relative to GO, but does not facilitate additional water uptake due to its bulky and hydrophobic triarylmethane framework that limits the available free volume for water molecules. By contrast, AZ1, the most planar intercalant, promoted tight π–π stacking and minimized free volume for hydration, yielding the smallest spacing. The ∆*d*/*d* value in Figure 1c (fractional difference in average *d*‐spacing between the wet and dry states) characterizes the swelling propensity, and follows a consistent trend in the following order: GO‐AZ2 > GO > GO‐TZ1 > GO‐TM1 >GO‐AZ1. The particularly low ∆*d*/*d* values for TM1 and AZ1 highlight their ability to stabilize GO galleries against swelling, suggesting the possibility of accessing ″deep NF″ properties.

**FIGURE 2:**
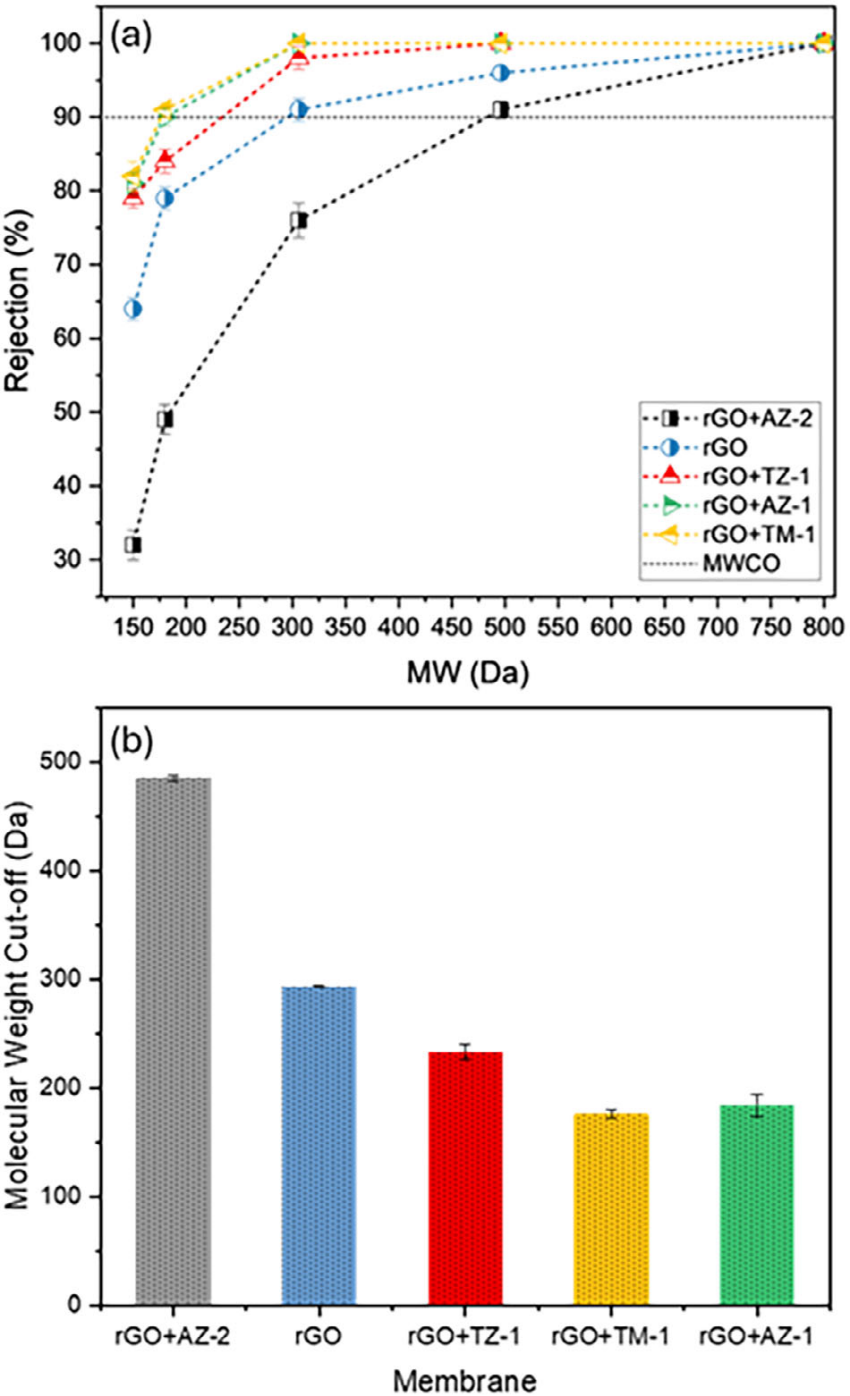
(a) Rejection trends of rGO intercalated membranes as a function of molecular weight of the permeating species (neutral molecules and charged dye molecules). (b) Molecular weight cut‐off for rGO intercalated membranes.

Next, we conducted permeation measurements on the compacted rGO and rGO‐X membranes to gain a detailed understanding of the structure‐property relationships. Figure 2a,b depicts the molecular weight cut‐offs (MWCOs) of all five membranes using molecules (cationic, neutral, and anionic) with molecular masses from 150–800 Da (Table S1). The net charge of these species (excluding neutral molecules) is assumed to have minimal influence over the rejection behavior since they are large molecules and are mostly rejected through sieving effects. The MWCO trend: rGO‐AZ2 > rGO > rGO‐TZ1 > rGO‐TM1 ∼ rGO‐AZ1, is broadly consistent with ∆*d*/*d* spacing trends. The MWCO values for rGO‐AZ1 (184±11 Da) and rGO‐TM1 (176±4 Da) are much lower than any GO or rGO NF membrane reported to our knowledge, highlighting the excellent potential of these intercalated membranes to be tuned toward “deep NF” (<200 Da) while maintaining permeability. All the membranes operate in the NF pressure range (< 50 bar), much lower than RO.

**FIGURE 3:**
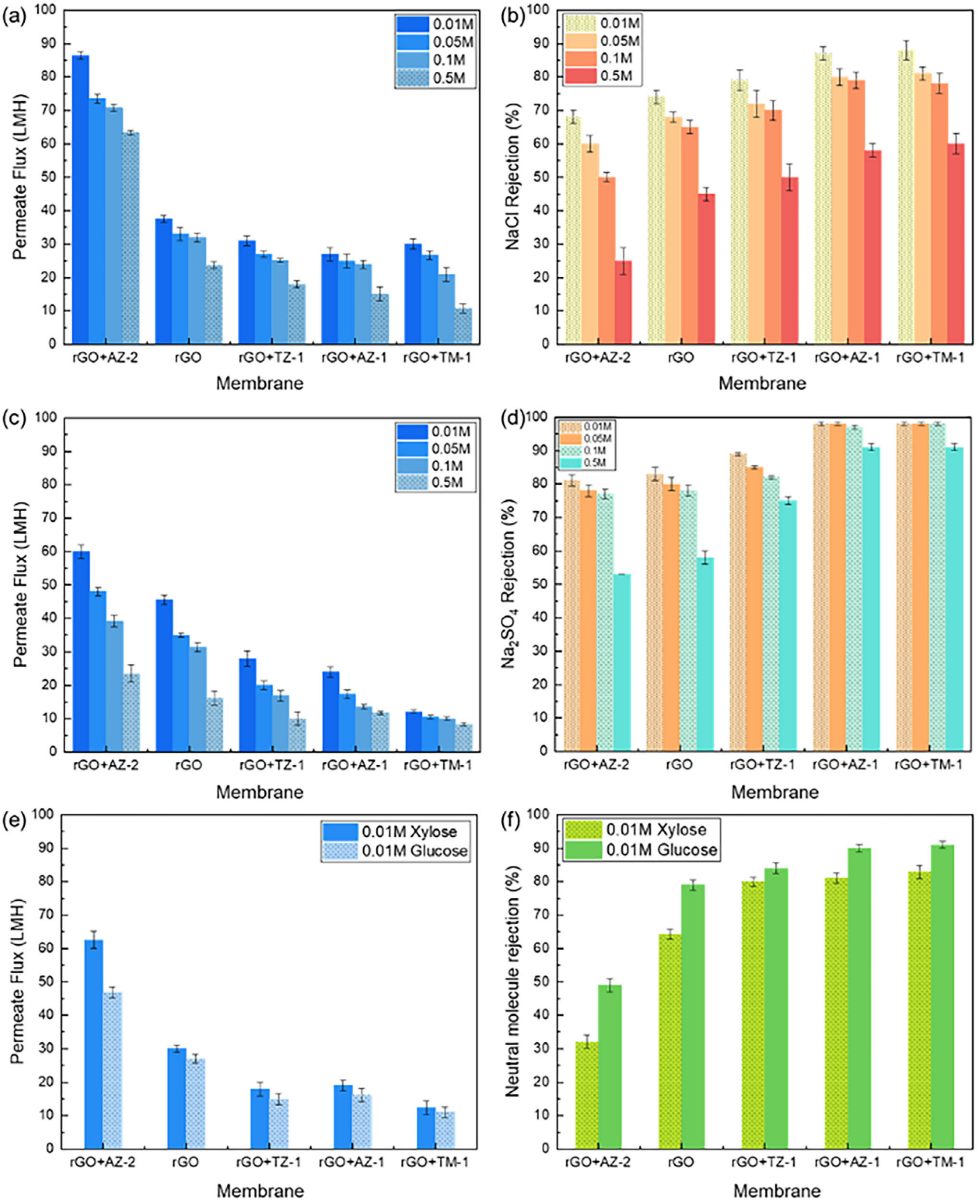
Rejection and flux trends of rGO‐X membranes at various salt concentrations (0.01–0.5 m) as a function of rGO intercalated membranes for (a, b) NaCl, (c, d) Na_2_SO_4_, and (e, f) neutral molecules (glucose & xylose).

Figure 3a‐f shows the permeate fluxes and solute rejections of the five membranes at R.T. and 50 bar, for four different aqueous feeds over a range of solute concentrations: monovalent salt (NaCl, 0.01–0.5 m), divalent salt (Na_2_SO_4_, 0.01–0.5 m), and neutral solutes (glucose and xylose, 0.01 m each). The bare rGO and rGO‐AZ2 membranes showed systematically higher permeate fluxes across both the salt and neutral solute feed streams, and also showed systematically lower solute rejections. Furthermore, the salt rejections dropped more significantly at higher feed concentrations for both NaCl and Na_2_SO_4_, due to the screening of the zeta potential repulsion effect at higher salt concentrations [66]. Under these conditions, size sieving of hydrated ions dominates over electrostatic effects, with chloride rejected less efficiently than sulfate.

**FIGURE 4:**
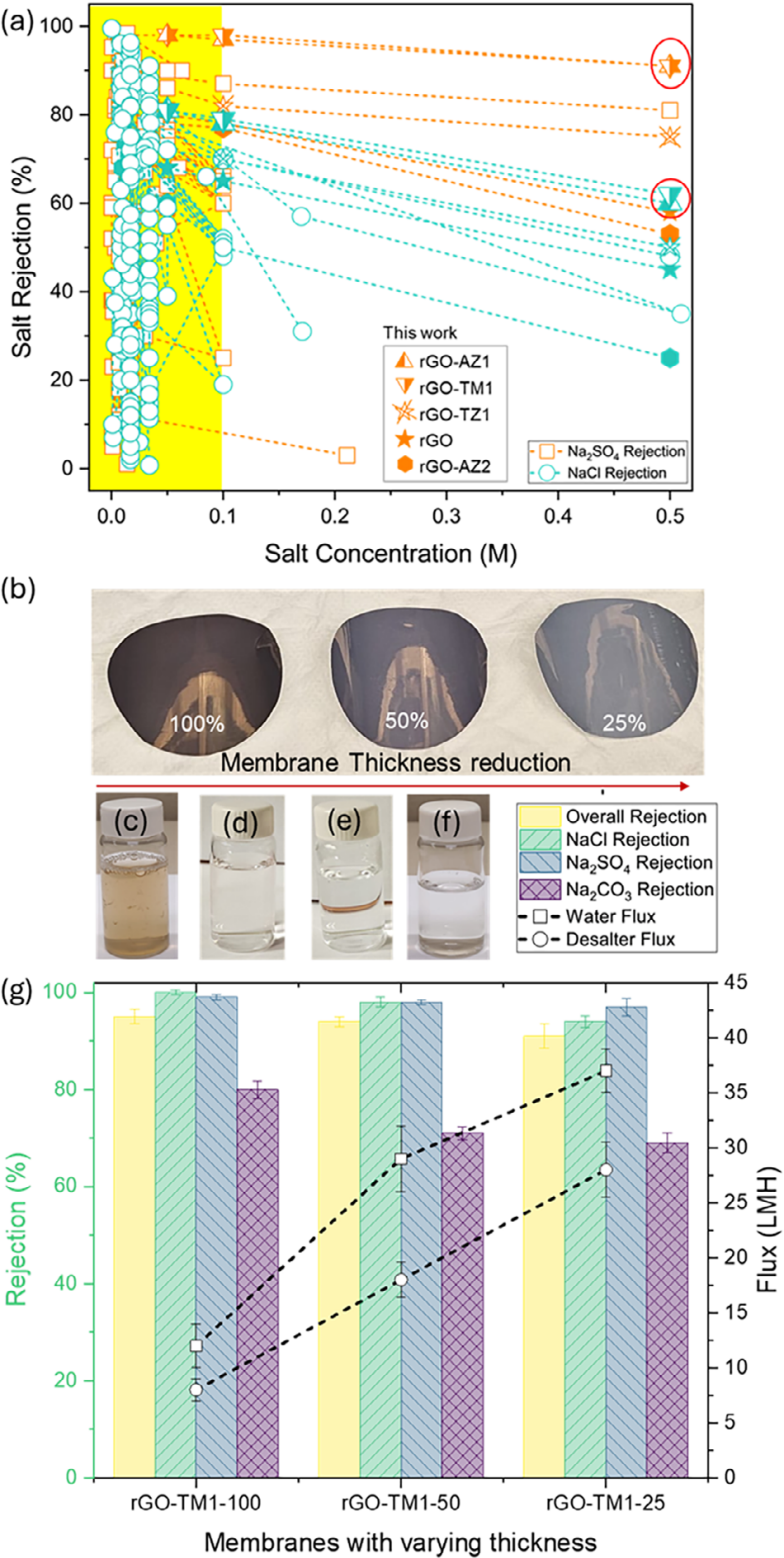
(a) Literature review of NaCl and Na_2_SO_4_ rejection as a function of feed concentration. Most prior studies (highlighted in yellow) focus on low concentrations, whereas the membranes developed in this work show superior rejections across a broader and more realistic salinity range. (b) Photograph of rGO‐TM1 membranes with varying thicknesses from 140 nm to 35 nm. Bright patterns appearing in the membrane samples are only an effect of overhead lighting reflections. (c) Wastewater feed (Desalter effluent) used for NF processing. (d‐f) Photographs of permeate samples obtained using membranes with 100%, 50%, and 25% relative thickness, respectively. (g) Overall inorganic rejection, detailed ion‐specific rejection, and permeate flux (pure water and desalter wastewater) of rGO‐TM1 membranes with varying thickness.

The hydrated monovalent chloride ion radius is relatively small (< 0.4 nm, < 400 kJ/mol hydration energy) and can partially shed its hydration shell to pass through confined galleries. The hydrated divalent sulfate ion radius is larger (>0.7 nm, >1000 kJ/mol hydration energy) and retains a strongly bound hydration shell with up to ∼15 water molecules, thus hindering permeation [46]. This difference explains the consistently higher rejections observed for sulfate compared to chloride, in agreement with Donnan equilibrium and Nernst‐Planck transport predictions. The low rejections of the two neutral solutes (i.e., charge repulsion effects switched off) also corroborate the above discussion. These results are fully consistent with the findings regarding rGO and rGO‐AZ2 membranes shown in Figures 1 and 2. On the other hand, the rGO‐TZ1, rGO‐AZ1, and rGO‐TM1 intercalated membranes demonstrated high solute rejections, albeit with lower (but substantial) fluxes in the range of 10–30 LMH. Divalent salt rejections are high (90–98%) at all concentrations, and the small (150–180 Da) neutral solute rejections approach 90%. Remarkably, the monovalent salt rejections are significantly increased (in, and remain> 50% even at high concentrations of 0.5 m. Once again, these results are fully consistent with the findings in Figures 1 and 2 regarding rGO‐TZ1, rGO‐AZ1, and rGO‐TM1 membranes shown. Figure 4a shows a summary of monovalent and divalent salt rejections of rGO‐based nanofiltration membranes from literature, as well as the present membranes, as a function of the salt concentration. This is a significant update from our previous summary [50], and includes new literature data over the last four years (Table S2). The earlier literature data are also retained, and the relevant references are cited [26, 42, 50, 65, 66, 67, 68, 69, 70, 71, 72, 73, 74, 75, 76, 77, 78, 79, 80, 81, 82, 83, 84, 85, 86, 87, 88, 89, 90, 91, 92, 93, 94, 95, 96, 97, 98, 99, 100, 101, 102, 103, 104, 105, 106, 107, 108, 109, 110, 111, 112, 113, 114, 115, 116, 117, 118, 119]. The present rGO‐TM1 and rGO‐AZ1 nanofiltration membranes demonstrate excellent salt rejections, over a large range of salt concentrations (up to 0.5 M) that cover many realistic nanofiltration application areas.

These findings are further illustrated in the context of an industrial application, i.e., processing of petroleum desalter effluent. In the petroleum refinery, crude oil is first contacted with water to remove salts/inorganics, and the aqueous phase is separated electrostatically, creating a wastewater effluent. This single operation constitutes a large fraction (10%) of water use in a typical refinery [120]. To illustrate the use of the present membranes under realistic conditions, we measured the cross‐flow NF performance of our selected candidate membrane (rGO‐TM1) with desalter wastewater feed received from a refinery in the USA. Table S3 shows a summary chemical analysis of the wastewater feed and effluent obtained from the abovementioned rGO‐TM1 membranes. This wastewater is rich in salts and contains suspended solids, dissolved organics, and entrained crude oil droplets. The presence of residual crude oil and organics makes conventional polymeric NF membranes susceptible to degradation or failure, whereas rGO materials are not significantly affected.

Figure 4b shows the visual images of three rGO‐TM1 membranes fabricated with 100%, 50%, and 25% relative quantity of the rGO‐TM1 suspension, thus varying the membrane thickness. Here, “100%” refers to the membrane formulation described previously in this report (∼130 nm thickness), and the “50%” and “25%” variants correspond to thinner (∼70 nm and ∼40 nm, respectively) membranes.

Figure 4c shows a photograph of the refinery desalter feed stream, and Figure 4d–f shows the permeate produced using 100%, 50%, and 25% rGO‐TM1 membranes. As shown in Figure 4g and Table S3, the rGO‐TM1 membrane achieved high solute rejections (as well as ∼100% rejection of entrained hydrocarbons and suspended solids). The rGO‐TM1‐100%, rGO‐TM1‐50%, and rGO‐TM1‐25% membranes produced permeates with a total solids content of 0.3 g/L (300 ppm), 0.45 g/L, and 0.51 g/L, respectively, from the 2.96 g/L feed stream, at fluxes of 8±1 LMH, 18±2 LMH, 28±3 LMH, at 50°C and 50 bar (Figure 4g). Notably, the NaCl rejection was comparable to that of Na_2_SO_4_, consistent with their similarly low feed concentrations (∼0.0065 m and ∼0.0074 m, respectively). In contrast, the rejection of carbonate salts was relatively lower, which can be attributed to their higher feed concentration (∼0.0135 m), nearly twice that of NaCl and Na_2_SO_4_. The permeate stream is suitable for reuse in desalting operations or elsewhere in the refinery (e.g., cooling towers).

To further evaluate the fouling resistance and longer‐term operational stability of the rGO‐TM1 membrane, a new batch of desalter wastewater from the refinery (Table S4) was processed through the rGO‐TM1 membrane continuously for a total time of ∼380 h (Figure S17). Since refinery desalter wastewater has large fluctuations in composition, each new batch received is expected to be different. In comparison to the feed used for the data in Figure 4g, this longer‐term study used a feed with much higher salinity (NaCl concentration 12 times higher, Table S4). This would result in a somewhat lower overall rejection, which is still much higher than previous literature (as shown in Figure 4a). The flux profile (Figure S17) shows that the permeate flux stabilized to ∼9 LMH during the initial hours of operation, due to the expected formation of a reversible concentration polarization layer including deposition of soluble organic/inorganic species on the membrane surface. Similar initial flux stabilization behavior is well known for NF membranes, including graphene‐based membranes [45]. Subsequently, the membrane maintained a steady‐state flux and overall rejection of ∼9 LMH & 70%, respectively. After the completion of Cycle 1, the membrane was cleaned in situ using a pH 12 NaOH solution, consistent with industrial membrane operations. This step fully restored the initial water flux, i.e., flux recovery ratio (FRR) of 100%, confirming that the accumulated surface species were predominantly reversible. The desalter water was then reintroduced (Cycle 2), and the membrane again reached the same steady‐state flux and overall rejection. Overall, these results demonstrate that the rGO‐TM1 membranes are robust and resistant to irreversible fouling. They can be effectively regenerated by intermittent cleaning consistent with industrial practice.

**FIGURE 5:**
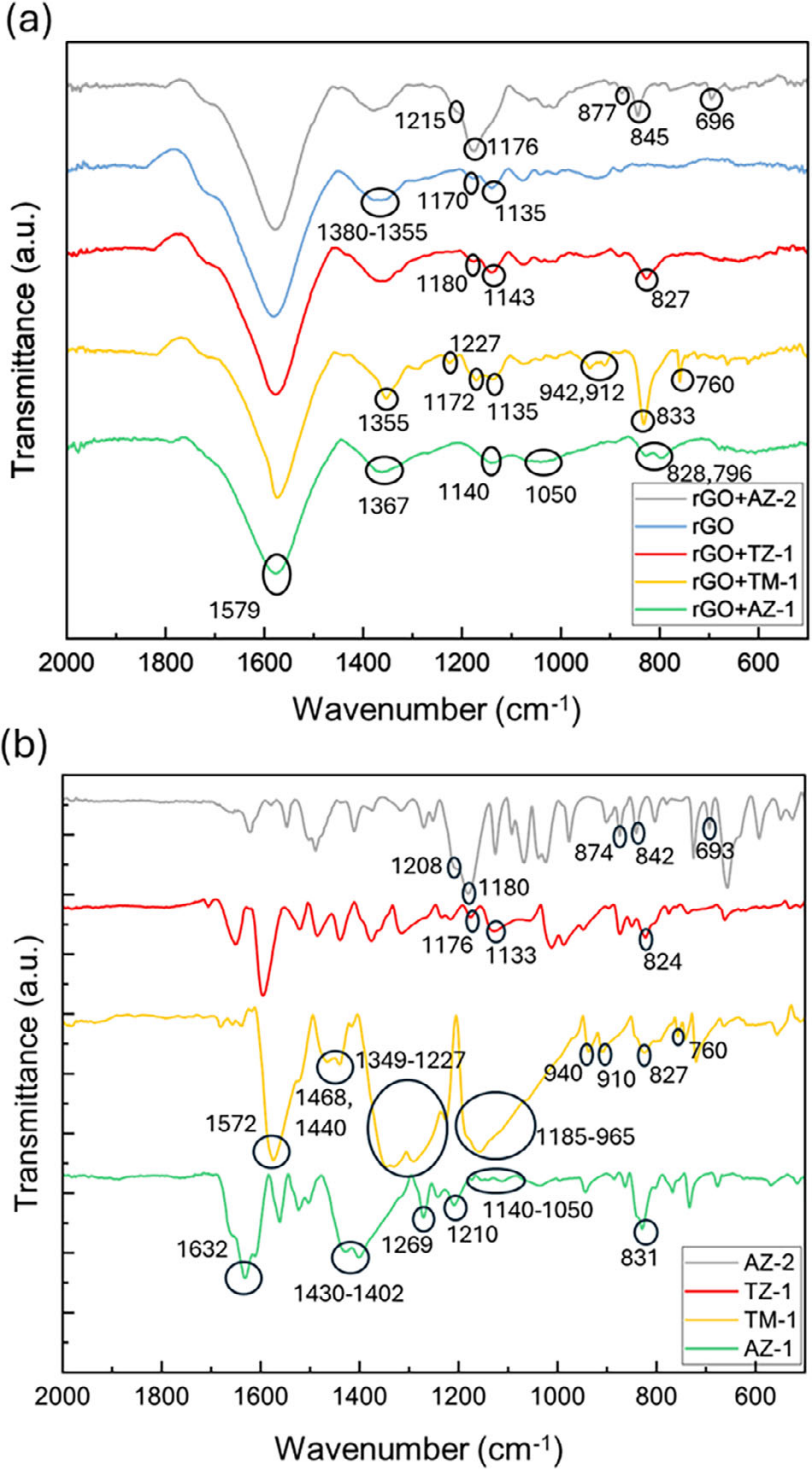
ATR‐FTIR spectra of (a) rGO and rGO‐intercalated free‐standing films, and (b) the corresponding powder intercalants.

Figure 5 shows ATR‐FTIR spectra obtained from five free‐standing (∼5 µm thick) rGO and rGO‐X membrane samples. The spectral features originate solely from the membrane material due to the absence of the underlying polymeric (PES) substrate. The full range ATR‐FTIR spectra are shown in Figure S18, and Figure 5a shows finer detail in the 2000–500 cm^−1^ region. Based on the known structures of rGO nanosheets and the four intercalants, the assignments of vibrational bands across the five membranes are listed in Table S5. The FTIR spectrum of the free‐standing rGO film (Figure S19a,b; Table S4) reveals the characteristic vibrational bands associated with residual oxygenated functional groups [121]. A broad absorption band around 3400 cm^−1^ corresponds to ─OH stretching vibrations, consistent with hydroxyl and carboxyl moieties. The weak band near 2920–2850 cm^−1^ is assigned to C─H stretching from aliphatic residues. A distinct peak at ∼1720 cm^−1^ indicates C═O stretching, attributable to aldehyde, carboxylic acid, or ketone functionalities, while the band at ∼1585 cm^−1^ corresponds to C═C stretching of *sp* ^2^‐hybridized domains. The region between 1350–1380 cm^−1^ is dominated by C─H and O─H bending, and multiple bands in the 1130–1070 cm^−1^ range are due to C─O stretching vibrations (Table S4). Compared to GO, the rGO shows a significant decrease in oxygen‐containing groups [122]. In particular, the diminished intensity of the C═O and C─O bands confirms partial removal of carbonyl, hydroxyl, and epoxide functionalities upon chemical reduction. All the intercalated rGO‐X films display the characteristic vibrations of rGO as shown in Figures S18 and S19. Several new peaks also appear, and are assigned to the intramolecular vibrations of the intercalants. This is verified by the corresponding powder ATR‐FTIR spectra of the intercalants (Figure 5b), with slight peak shifts due to differences in local environment between the powder state and the rGO interlayer space. Together with the FTIR and fluorescence data, it is concluded that intercalation does not lead to new chemical bond formation, and that the intercalants bind strongly to the GO or rGO surfaces mainly by π–π/π‐cation interactions due to their polyconjugated electronic structure.

## 3 Conclusion

This study demonstrates that intercalation of reduced graphene oxide (rGO) membranes with polyconjugated organic molecules provides a versatile strategy to tune interlayer structure and separation performance. The results show that intercalant geometry, conjugation, and substituent chemistry collectively regulate gallery spacing, swelling propensity, and solute rejection, underscoring the interplay between steric effects, π–π stacking, and hydration‐driven expansion. Importantly, the intercalation approach allows rGO membranes to achieve molecular weight cut‐offs (MWCOs) approaching those of reverse osmosis, i.e., the “deep NF” regime. The present findings further indicate that AZ1 and TM1, despite differences in planarity, both possess extended polyconjugated frameworks that promote strong π–π interactions with rGO, contributing to reduced interlayer spacings. By contrast, AZ2 incorporates bulky, hydrophilic sulfonate groups that introduce steric and electrostatic effects, leading to weaker association with rGO surfaces and larger gallery spacings. These observations highlight the importance of both molecular geometry and substituent chemistry in governing intercalant packing and interlayer structure. Overall, the characterizations provide an initial framework for connecting intercalant structure to membrane performance. However, the underlying mechanisms of solute rejection remain complex, and more detailed investigations, combining molecular simulations with advanced in situ characterizations, are needed to fully elucidate intercalant orientation, distribution, and dynamic interactions within rGO galleries.

## Conflicts of Interest

The authors declare no conflict of interest.

## Supporting information




**Supporting File**: advs75034‐sup‐0001‐SuppMat.pdf.

## Data Availability

The data that support the findings of this study are available from the corresponding author upon reasonable request.
