# Supplementary material for: Reduced‐Graphene Oxide Nanofiltration Membranes Intercalated by Conjugated Polyaromatics: Towards High Monovalent Salt Rejections
Source: Adv Sci (Weinh). 2026 Mar 26;13(33):e23231. doi: 10.1002/advs.202523231 (PMC13271638; doi:10.1002/advs.202523231)
Supplement: Supplementary file 1 — Supporting File: advs75034‐sup‐0001‐SuppMat.pdf. [file ADVS-13-e23231-s001.pdf]

Reduced-Graphene Oxide Nanofiltration Membranes Intercalated by Conjugated Polyaromatics: Towards High Monovalent Salt Rejections

Muskan Sonker[a], Sankar Nair*[a,b]

[a] M. Sonker, Prof. Dr. S. Nair
School of Chemical & Biomolecular Engineering
Georgia Institute of Technology
311 Ferst Drive NW, Atlanta GA 30332, USA
E-mail: sankar.nair@chbe.gatech.edu

Table of Contents

Experimental Procedures **4**

Materials 4

Preparation of GO and rGO-X membranes 4

Characterization of Suspensions and Membranes 4

Permeation measurements 4

Results and Discussion

Supplementary figures

Figure S1. Three-dimensional molecular renderings of the intercalants (a) AZ1, (b) AZ2, (c) TZ1, and (d) TM1) generated using Molview, highlighting their structural geometries (Top view(I) and side view(II)) used in this study. 5

Figure S2. Visual appearance of the aqueous intercalant solutions at different concentrations and constant volume, and after addition of rGO suspension. (a) TZ1, (b) rGO+TZ1, (c) AZ1, (d) rGO+AZ1, (e) TM1, (f) rGO+TM1, (g) AZ2, and (h) rGO+AZ2. 6

Figure S3. UV-Vis absorbance spectra of the aqueous intercalant solutions and rGO-X suspension as shown in Figure S2. (a) TZ1, (b) TM1, (c) rGO+TZ1, (d) rGO+TM1. 7

Figure S4. UV-Vis absorbance spectra of the aqueous intercalant solutions and rGO-X suspension as shown in Figure S2. (a) AZ1, (b) AZ2, (c) rGO+AZ1, (d) rGO+AZ2. 8

Figure S5. (a-h) Deconvolution of TZ1 absorbance spectra by a set of Gaussian peaks, for each concentration shown in Figure S3. (i) Integrated areas of each of the three spectral peaks as a function of the TZ1 concentration. (j) Overall integrated area as a function of the TZ1 concentration 9

Figure S6. (a-h) Deconvolution of TM1 absorbance spectra by a set of Gaussian peaks (600nm~Monomer and 548nm~H-dimer), for each concentration shown in Figure S3. (i) Integrated areas of the two spectral peaks as a function of the TM1 concentration. (j) Overall integrated peak area as a function of TM1 concentration. (k) Proposed TM1 (crystal violet) dimer structure (adapted from Ref. 57). 10

Figure S7. (a-h) Deconvolution of AZ1 absorbance spectra by Gaussian peaks, for each concentration shown in Figure S4. (i) Integrated areas of the spectral peaks as a function of the AZ1 concentration. 11

Figure S8. (a-h) Deconvolution of AZ2 absorbance spectra by Gaussian peaks, for each concentration shown in Figure S4. (i) Overall integrated peak area as a function of AZ2 concentration. 12

Figure S9. Solution state fluorescence spectra of intercalant X and corresponding rGO-X suspension as a function of wavelength at increasing concentrations: (a) TZ1, (b) rGO+TZ1, (c) TM1, and (d) rGO+TM1. 13

Figure S10. Solution state fluorescence spectra of intercalant and rGO mixed intercalant as a function of wavelength at increasing concentration (a) AZ1, (b) rGO+AZ1, (c) AZ2, and (d) rGO+AZ2. 14

Figure S11. Integrated fluorescence emission peak areas for X intercalant solution and corresponding rGO-X suspension, as a function of total intercalant concentration (a) TZ1, (b) TM1, (c) AZ1, and (d) AZ2. The dashed lines are only a guide to the eye. 15

Figure S12. Adsorption isotherms for intercalant binding on rGO as calculated from the data in Figure S11 16

Figure S13. Top-view SEM images of (a) rGO, (c) rGO-TZ1, (c) rGO-TM1, (d) rGO-AZ1, and (d) rGO-AZ2 membranes. 17

Figure S14. Cross-section SEM images of (a) rGO, (c) rGO-TZ1, (c) rGO-TM1, (d) rGO-AZ1, and (d) rGO-AZ2 membranes. 18

Figure S15. Peak fitted solid-state UV-Vis absorbance spectra of intercalated rGO-X membranes: (a) rGO-TZ1, (b) rGO-TM1, (c) rGO-AZ1, and (d) rGO-AZ2. 19

Figure S16. Raw XRD patterns of the GO-X membranes for (a) dry and (b) wet compacted situations. 20

Figure S17. Filtration performance of rGO-TM1 membrane during two cycles of desalter wastewater treatment with intermediate cleaning. 20

Figure S18. ATR-FTIR spectra of rGO and rGO-X free-standing films over the full range (4000–500 cm^{-1}). 21

Figure S19. Detailed FTIR spectra of free-standing rGO films: (a-b) rGO, (c) rGO-TZ1, (d) rGO-TM1, (e) rGO-AZ1, and (f) rGO-AZ2. 22

Supplementary Tables

Table S1. Molecules used in molecular weight cut-off measurements. 23

Table S2. Numerical data corresponding to Figure 4a. 23-29

Table S3. Chemical analysis of the petroleum desalter wastewater feed stream used in this work, and the permeate obtained from varying thickness rGO-TM1 membranes. 29

Table S4.Chemical analysis of the petroleum desalter wastewater (B) feed stream used in the long-term study (Figure S17), and the corresponding steady state permeate obtained from the rGO-TM1 membrane. 29

Table S5. Assignments of vibrational bands observed in the FTIR spectra of rGO and intercalated rGO membranes. 30

Author Contributions 30

SUPPORTING INFORMATION

1. Experimental Procedures

1.1. Materials

Toluidine Blue O, Crystal Violet, Bismarck Brown Y, and Allura red AC were obtained from Sigma Aldrich and used as received. PES (Poly-ether sulphone) supports (30nm pore size) were obtained from Sterlitech. Desalter water was obtained from Phillips 66 and used as received.

1.2. Preparation of GO and rGO-X membranes

GO and rGO suspensions (1 g/L) were prepared using a modified Hummers method and thermal reduction via NaOH, as described in our previous work. The rGO-X membrane coupons with varying amounts of X (molecule/intercalant) (~9 wt% TZ-1 mol-wise (0.33 µmol)) were prepared by mixing reagent 'A' (1 mg/mL rGO solution) with varying amounts of reagent 'B' (0.5 mg/mL TBO), as detailed in our previous study. In this work, we used PES (30 nm effective pore size ultrafiltration (UF) membrane by Sterlitech) supports to fabricate the membranes. Following fabrication, the membrane coupons were allowed to air dry at room temperature for 24 hours.

The amount of X molecule loaded on each rGO-X membrane coupon was calculated based on the difference between the amount of X initially used for preparing the dispersion and the X remaining in the vacuum filtrate, as determined in our previous work using UV-Vis spectroscopy. For all the rGO-X membranes except AZ-2, all the molecules/intercalants initially added to the suspension were successfully coated on the membrane.

1.3. Characterization of Suspensions and Membranes

XRD spectra for GO-TBO membrane coupons were obtained using a Rigaku Miniflex XRD instrument. Liquid UV-Vis spectra of X and rGO-X suspensions at various concentrations were acquired using an Agilent 8453 UV-Vis spectrophotometer (Agilent Technologies). Fluorescence spectra for X and rGO-X aqueous dispersions were obtained using a Horiba FL3-21 fluorometer. UV-Vis absorbance spectra (400-800 nm) in the solid state for the rGO-X membrane coupons were obtained using a UV-Vis/NIR double beam spectrophotometer (Cary 5000), which employs a deuterium arc light and a tungsten halogen source to illuminate the sample with light spanning UV to near-IR, as detailed in our previous work. Surface zeta potential was measured at neutral pH and room temperature using a Zetasizer Nano ZS instrument. Standard nanospheres with a uniform size distribution (60± 4nm, 3000 Series Nanosphere size standards, Thermo Fisher Scientific) were utilized as tracer particles. Molecular structures were identified using a Thermo Nicolet 6700 Fourier Transform Infrared Spectrometer (FTIR) with a diamond crystal single-bounce attenuated total reflectance (ATR) attachment.

To analyze the desalter water components, the total suspended solids (TSS) were calculated by measuring the difference in weight of the PES support (~30 nm) before and after filtering the desalter water. The total solids (TS) in the desalter water sample were calculated by summing the total salts present and the amount of solids deposited on the PVDF substrate by vacuum filtering the desalter water sample. The total organic carbon (TOC) was measured using coulometry, and the anion concentration was measured using anion chromatography.

1.4. Permeation measurements

Salt permeation measurements were performed in a dead-end cell mode using high-pressure HP4750X Sterlitech stirred cells, which have a feed capacity of 300 ml and a maximum operating pressure of 2500 psi. The measurements were driven by pressurized N_2 gas cylinders at ambient temperature. Membrane coupons (47 mm in diameter) were placed at the bottom of the cell, supported by a ceramic holder. Permeation measurements were conducted at 50 bar and room temperature. The cells were continuously stirred at 340 rpm, corresponding to a Reynolds number of ~35000. The flux and rejection values were calculated using the following equations: $J = \Delta m/(\rho A \Delta t)$ and $R = (1-C_p/C_r)$ ×100. Here Δm is the amount of permeate collected, Δt is the time, ρ is the permeate density, A is the membrane area, C_p is the permeate concentration, and C_r is the retentate concentration. The desalter wastewater permeation was conducted in a Sterlitech CF047 stainless-steel cross-flow cell (channel height = 0.19 cm; half channel width = 2.35 cm) operated at a feed flow rate of 190 mL min^{-1}. Using the manufacturer-provided channel geometry, the corresponding cross-flow velocity was 7.10 cm s^{-1}. The hydraulic diameter of the channel was calculated as 0.352 cm, giving a Reynolds number of ~280 (ρ = 997 kg m^{-3}, μ = 0.89 mPa·s at room temperature), confirming laminar flow during all experiments.

2. Supporting Figures

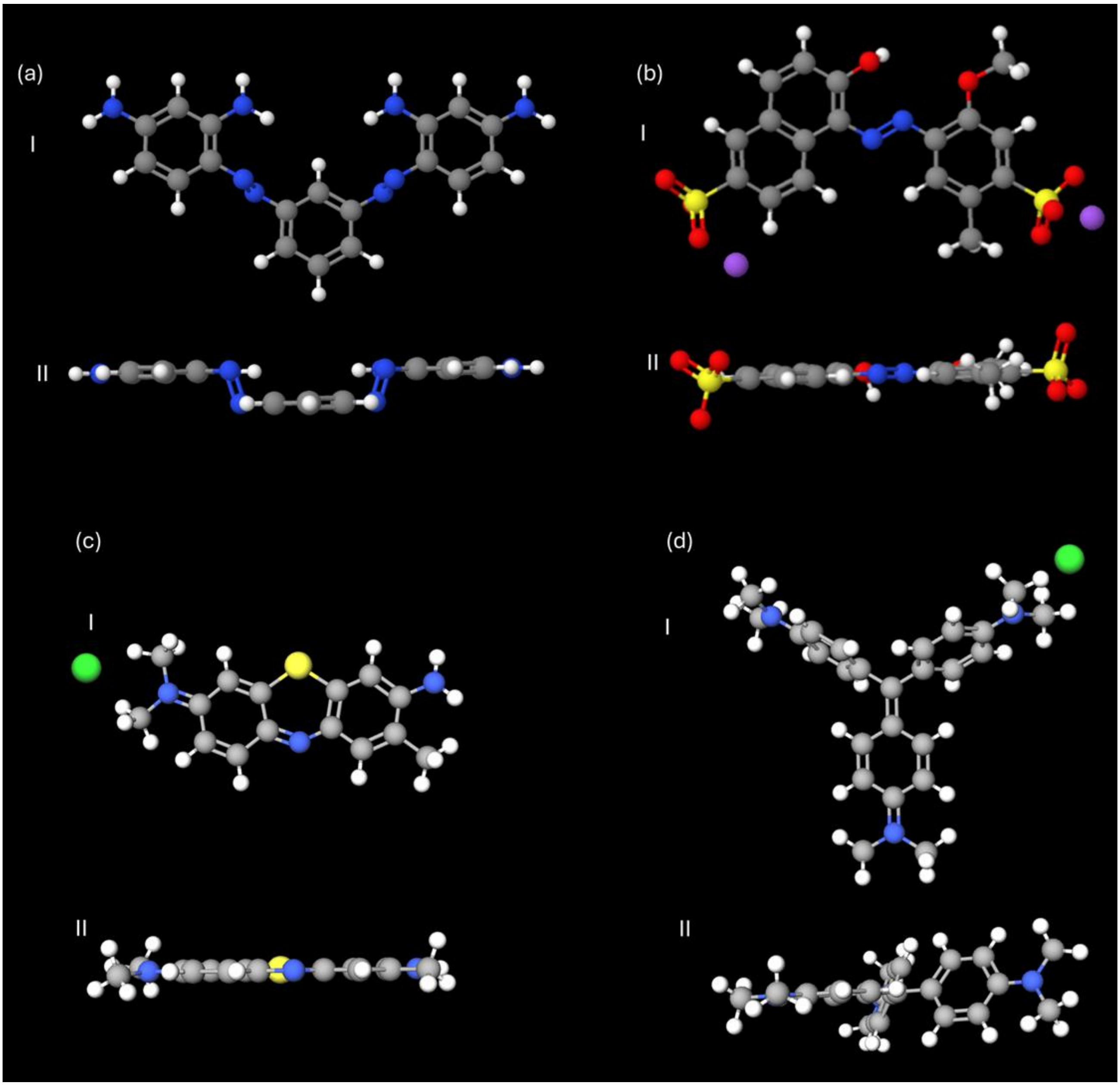

Figure S1. Three-dimensional molecular renderings of the intercalants (a) AZ1, (b) AZ2, (c) TZ1, and (d) TM1) generated using Molview, highlighting their structural geometries (Top view(I) and side view(II)) used in this study. Atom colors follow the CPK (Corey-Pauling-Koltun) scheme as used in MolView: Carbon-grey; Hydrogen-white; Nitrogen-blue; Oxygen-red; Sulfur-yellow; Chlorine-green; Sodium-purple.

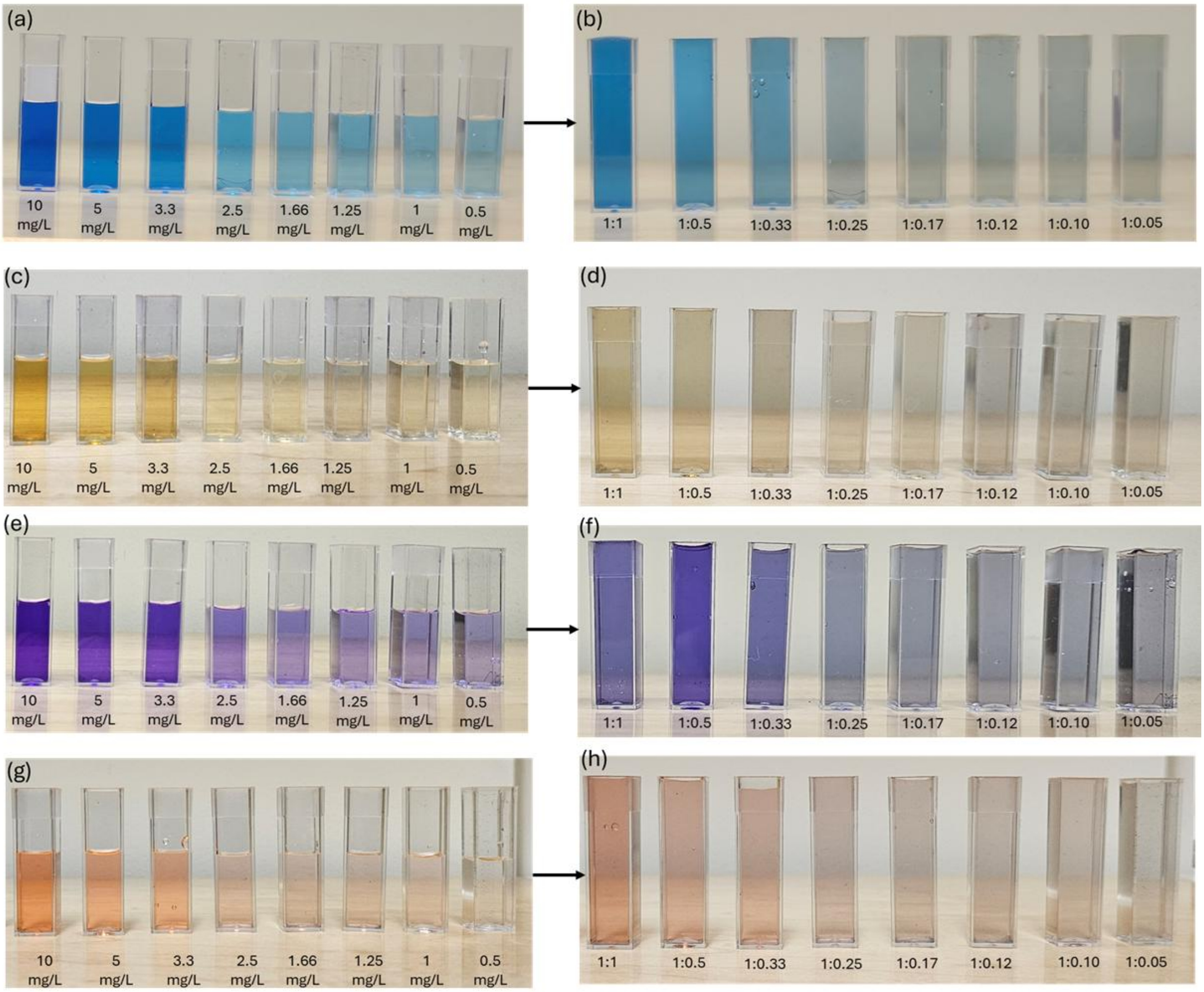

Figure S2. Visual appearance of the aqueous intercalant solutions at different concentrations and constant volume, and after addition of rGO suspension. (a) TZ1, (b) rGO+TZ1, (c) AZ1, (d) rGO+AZ1, (e) TM1, (f) rGO+TM1, (g) AZ2, and (h) rGO+AZ2. For the suspensions (b, d, f, h), the rGO:X mass ratios in the suspensions are 1:1, 1:0.5, 1:0.33, 1:0.25, 1:0.17, 1:0.12, 1:0.10, 1:0.05 where X = intercalants. The final rGO concentration is 5 mg/L in all the suspensions.

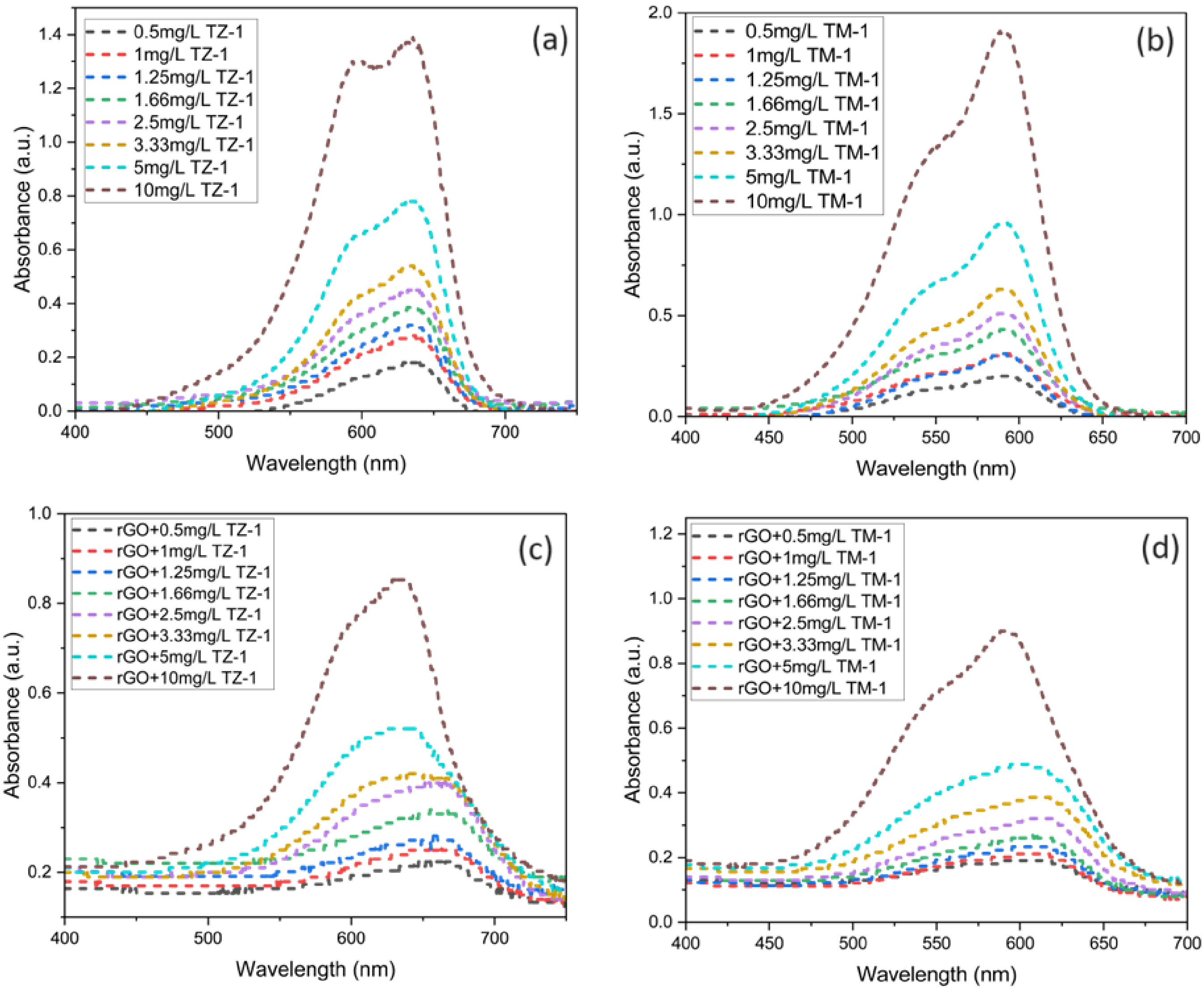

Figure S3. UV-Vis absorbance spectra of the aqueous intercalant solutions and rGO-X suspension as shown in Figure S2. (a) TZ1, (b) TM1, (c) rGO+TZ1, (d) rGO+TM1.

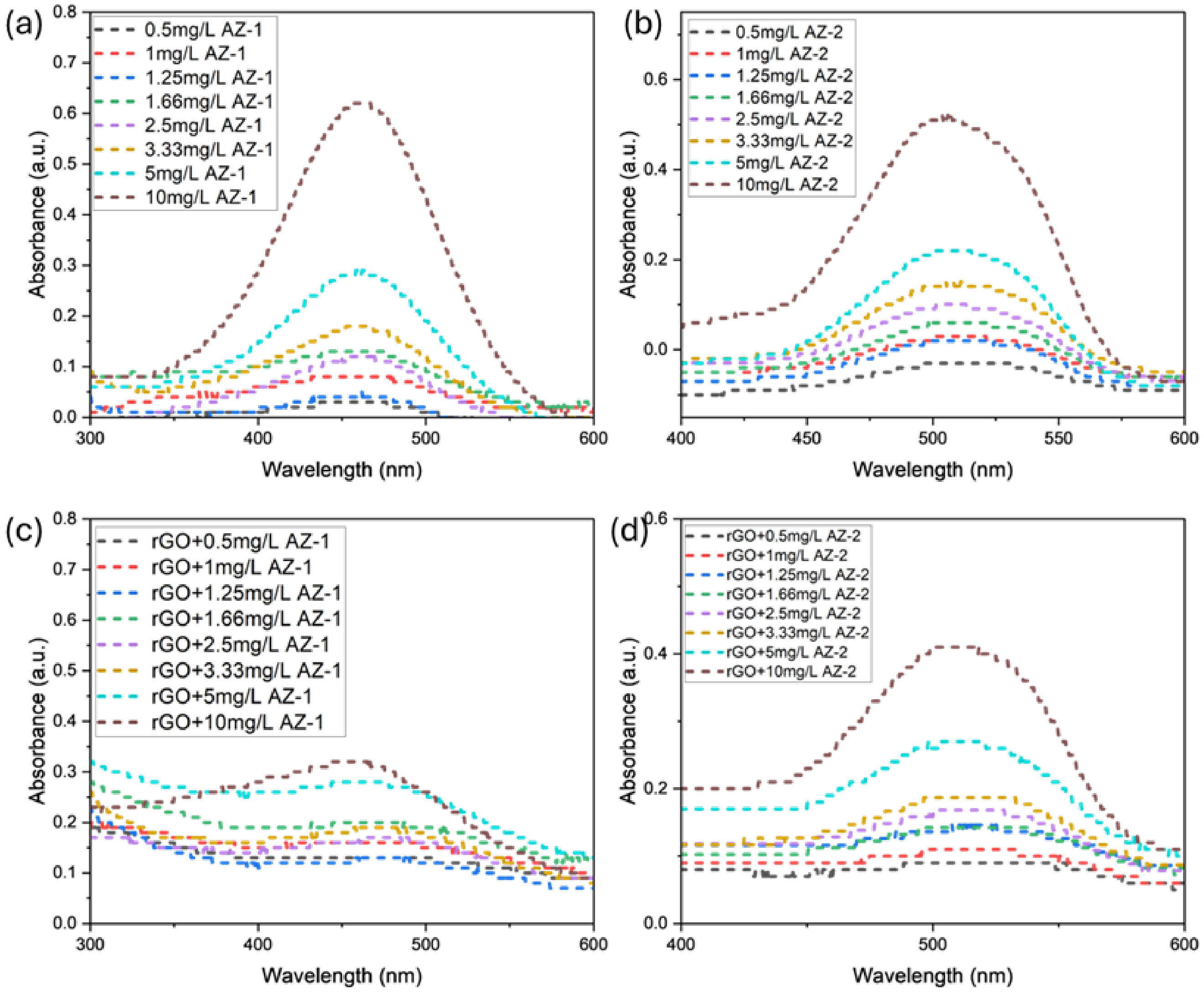

Figure S4. UV-Vis absorbance spectra of the aqueous intercalant solutions and rGO-X suspension as shown in Figure S2. (a) AZ1, (b) AZ2, (c) rGO+AZ1, (d) rGO+AZ2.

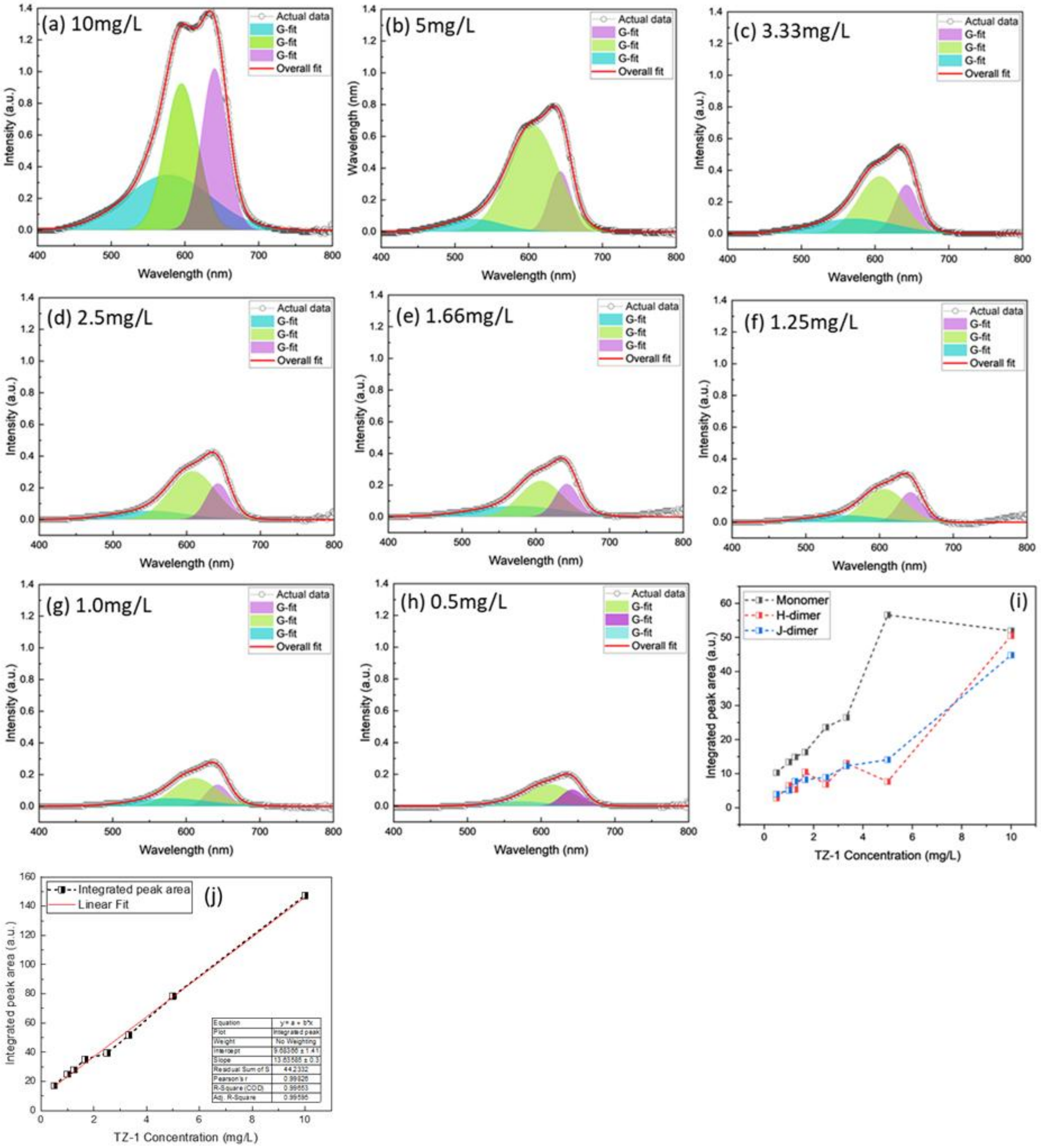

Figure S5. (a-h) Deconvolution of TZ1 absorbance spectra by a set of Gaussian peaks, for each concentration shown in Figure S3. (i) Integrated areas of each of the three spectral peaks as a function of the TZ1 concentration. (j) Overall integrated area as a function of the TZ1 concentration

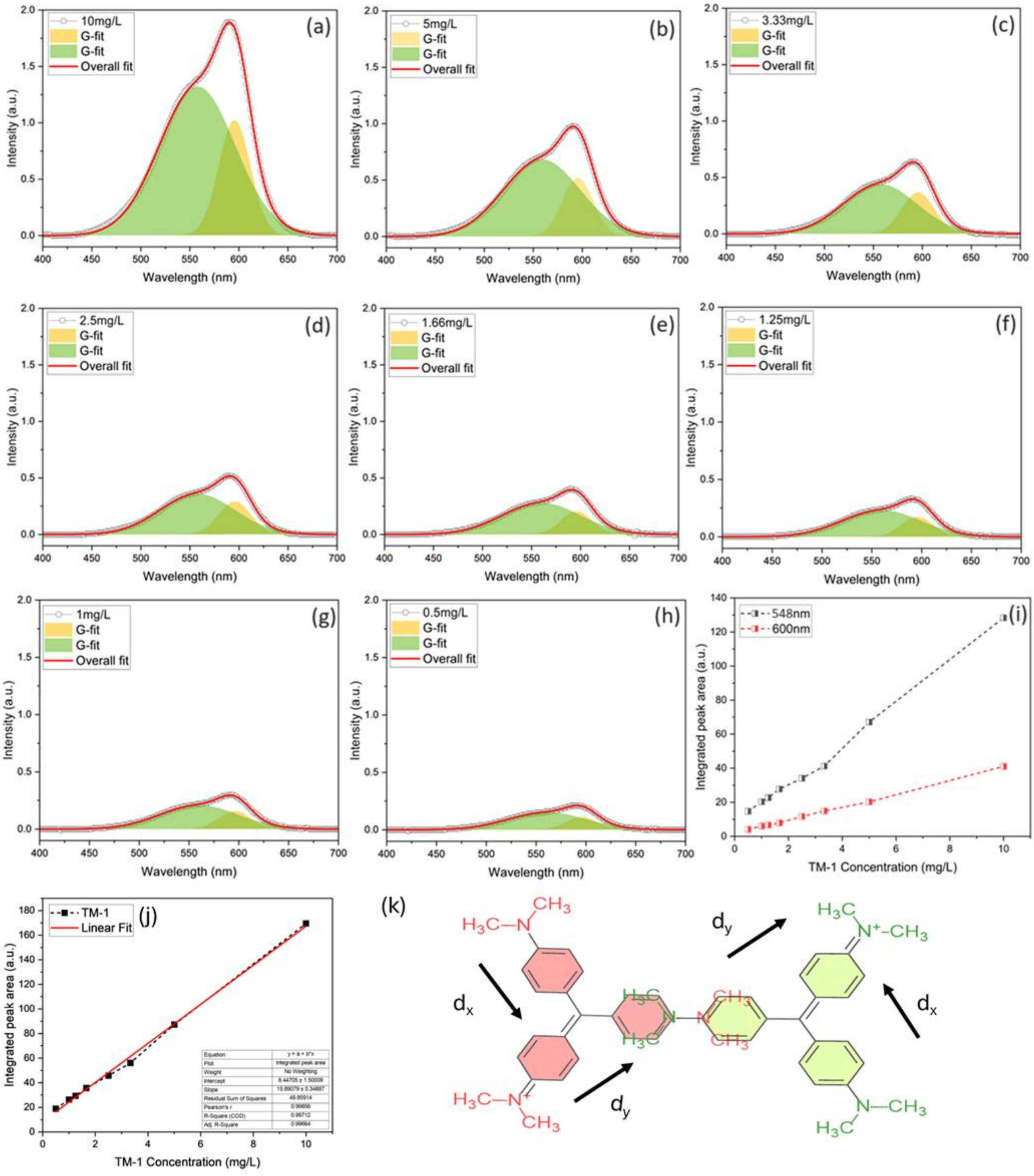

Figure S6. (a-h) Deconvolution of TM1 absorbance spectra by a set of Gaussian peaks (600nm~Monomer and 548nm~H-dimer), for each concentration shown in Figure S3. (i) Integrated areas of the two spectral peaks as a function of the TM1 concentration. (j) Overall integrated peak area as a function of TM1 concentration. (k) Proposed TM1 (crystal violet) dimer structure (adapted from Ref. 57).

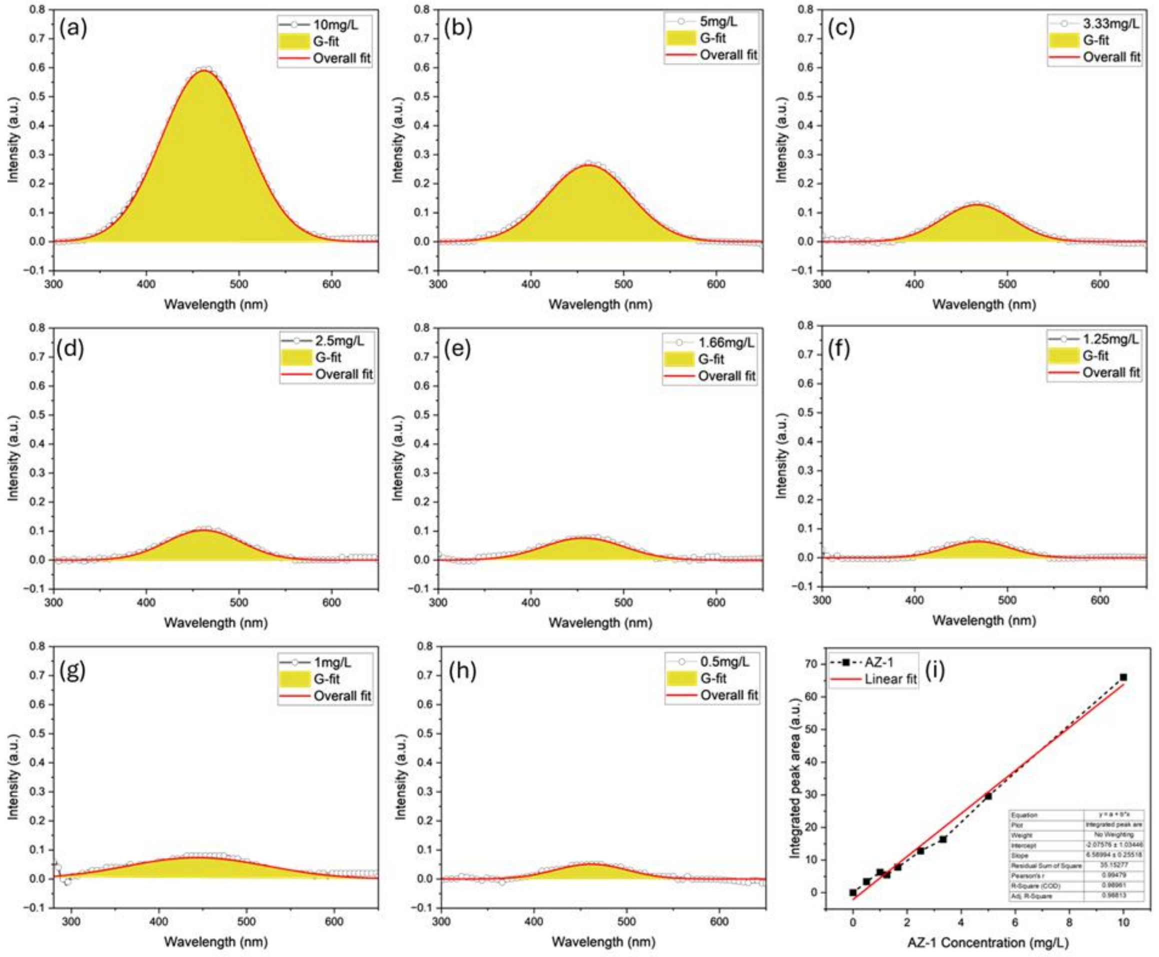

Figure S7. (a-h) Deconvolution of AZ1 absorbance spectra by Gaussian peaks, for each concentration shown in Figure S4. (i) Integrated areas of the spectral peaks as a function of the AZ1 concentration.

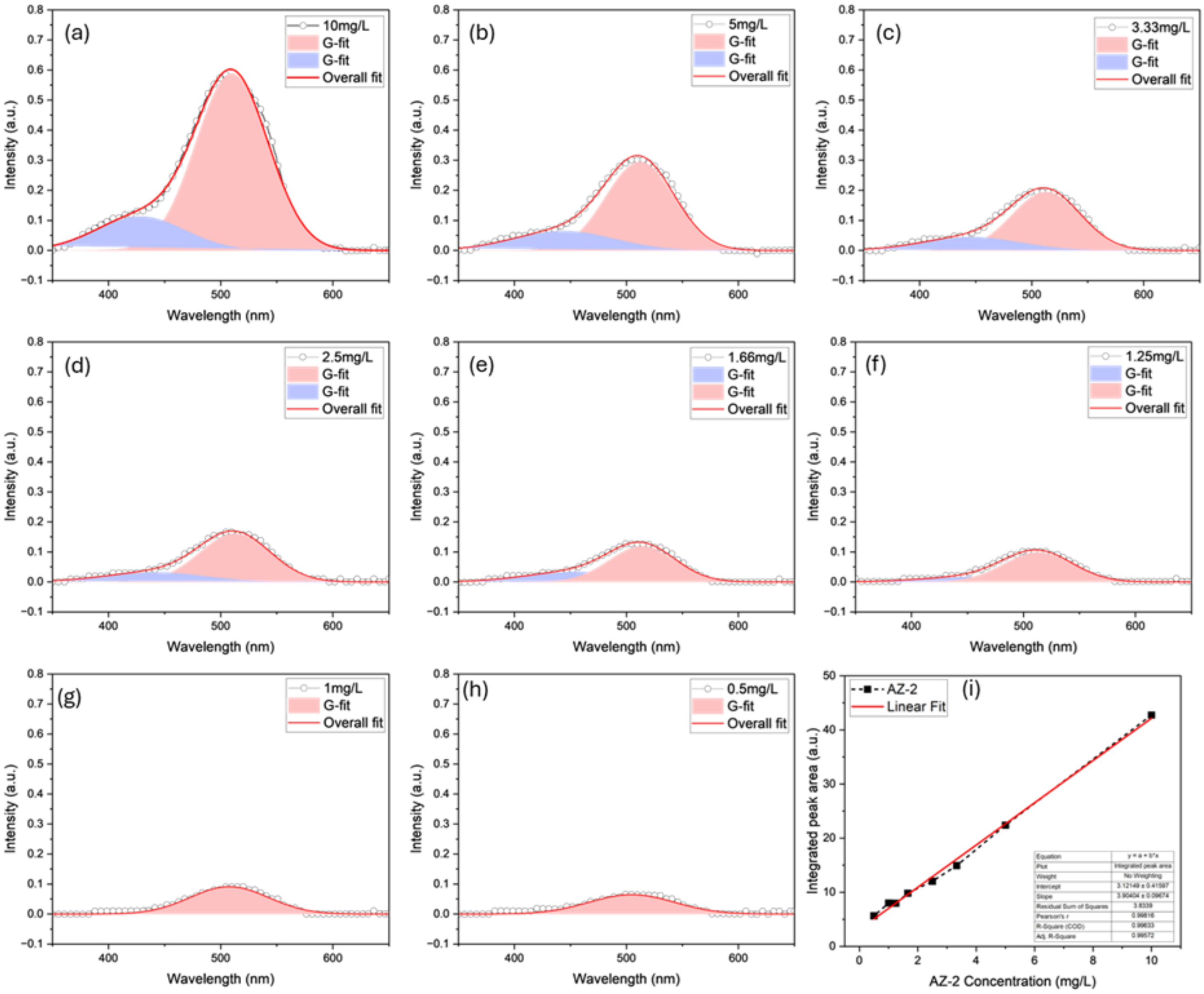

Figure S8. (a-h) Deconvolution of AZ2 absorbance spectra by Gaussian peaks, for each concentration shown in Figure S4. (i) Overall integrated peak area as a function of AZ2 concentration.

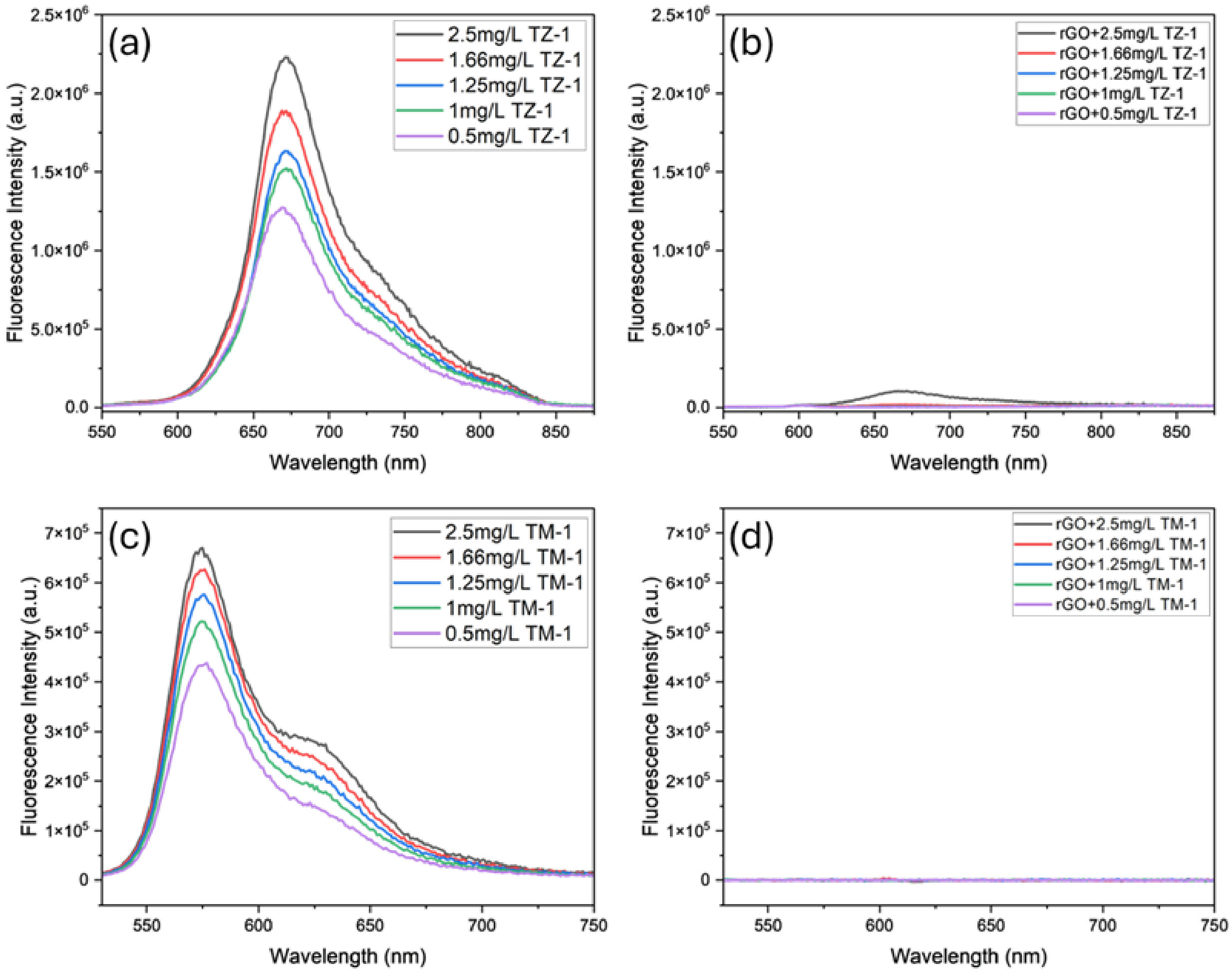

Figure S9. Solution state fluorescence spectra of intercalant X and corresponding rGO-X suspension as a function of wavelength at increasing concentrations: (a) TZ1, (b) rGO+TZ1, (c) TM1, and (d) rGO+TM1.

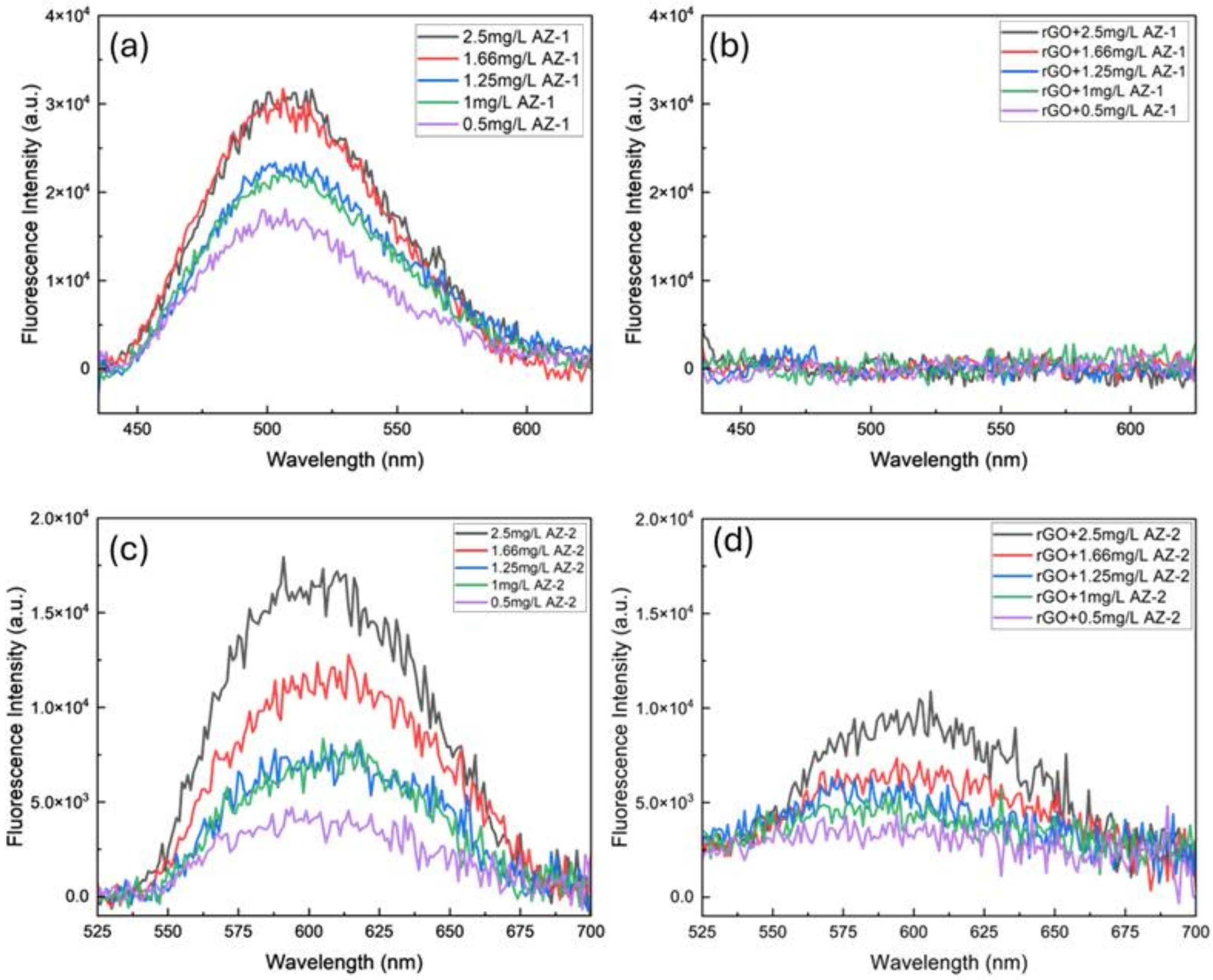

Figure S10. Solution state fluorescence spectra of intercalant and rGO mixed intercalant as a function of wavelength at increasing concentration (a) AZ1, (b) rGO+AZ1, (c) AZ2, and (d) rGO+AZ2.

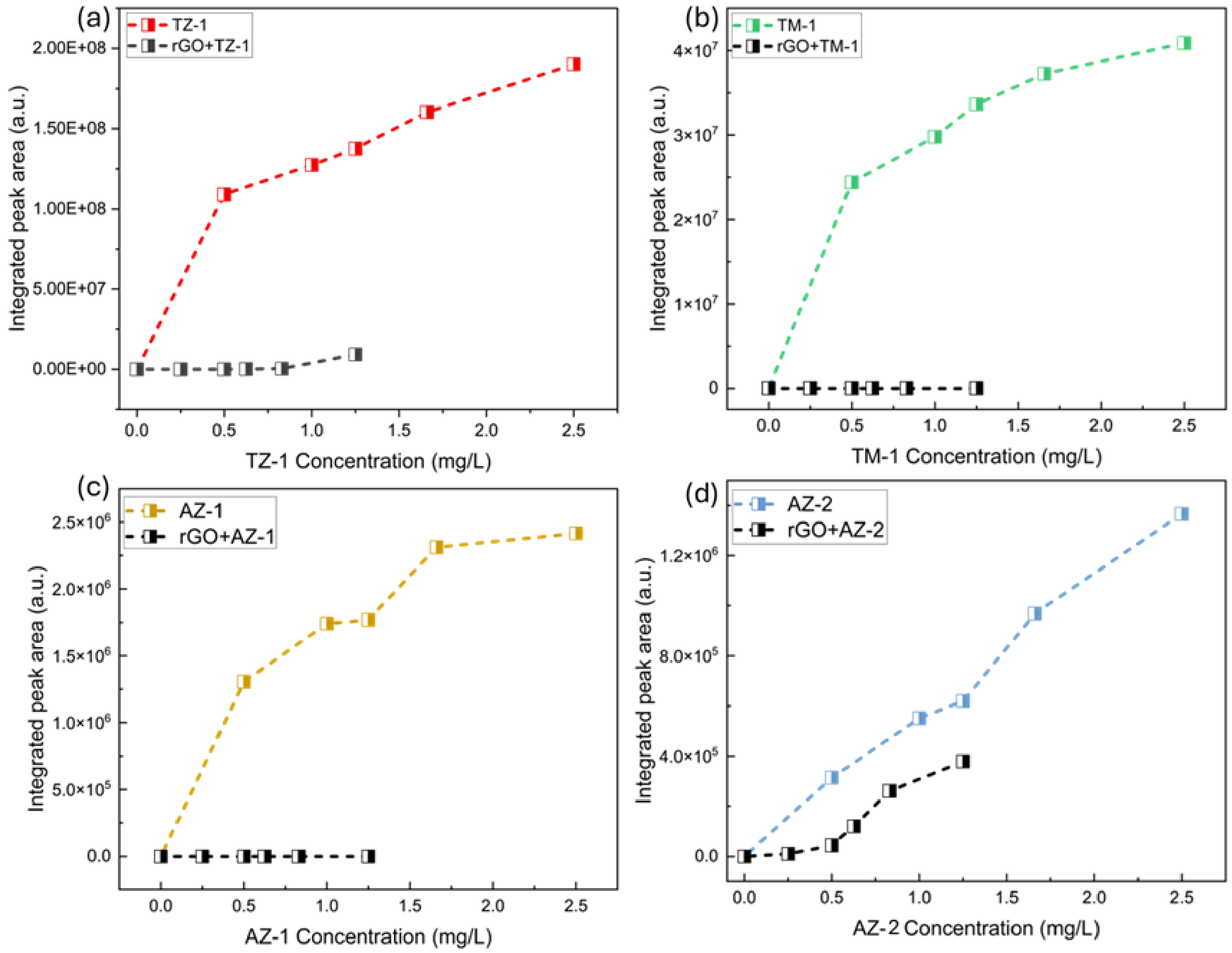

Figure S11. Integrated fluorescence emission peak areas for X intercalant solution and corresponding rGO-X suspension, as a function of total intercalant concentration (a) TZ1, (b) TM1, (c) AZ1, and (d) AZ2. The dashed lines are only a guide to the eye.

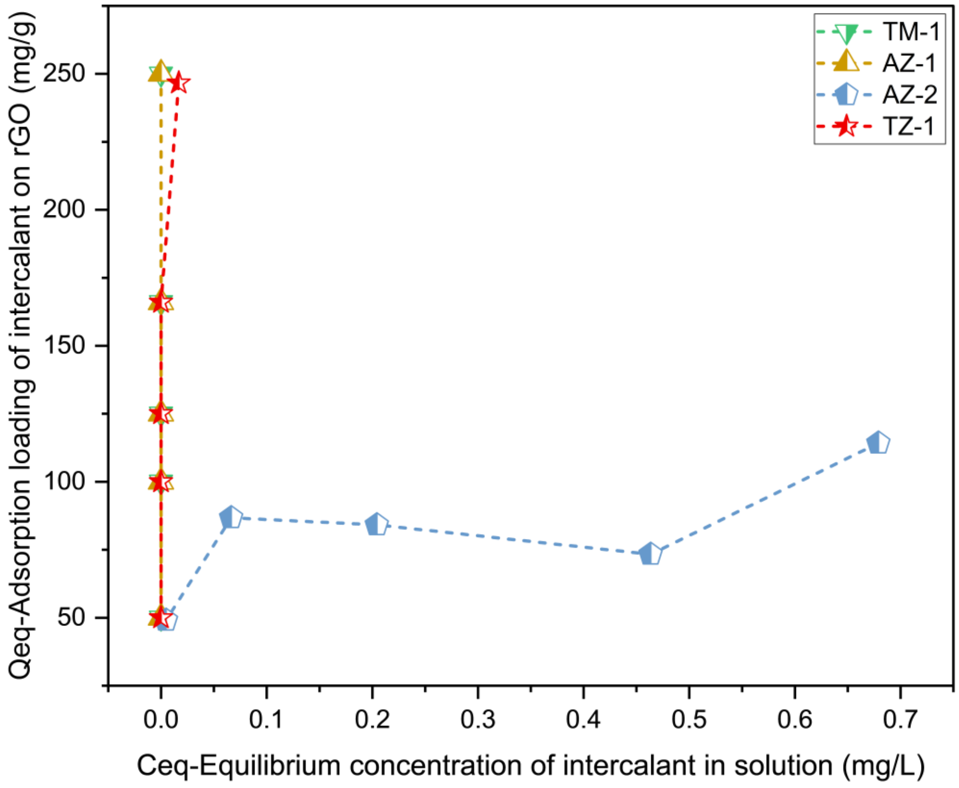

Figure S12. Adsorption isotherms for intercalant binding on rGO as calculated from the data in Figure S11. Here, Q_{eq} is the adsorption loading of the molecule on the rGO surface, and C_{eq} is the equilibrium concentration of dissolved intercalant.

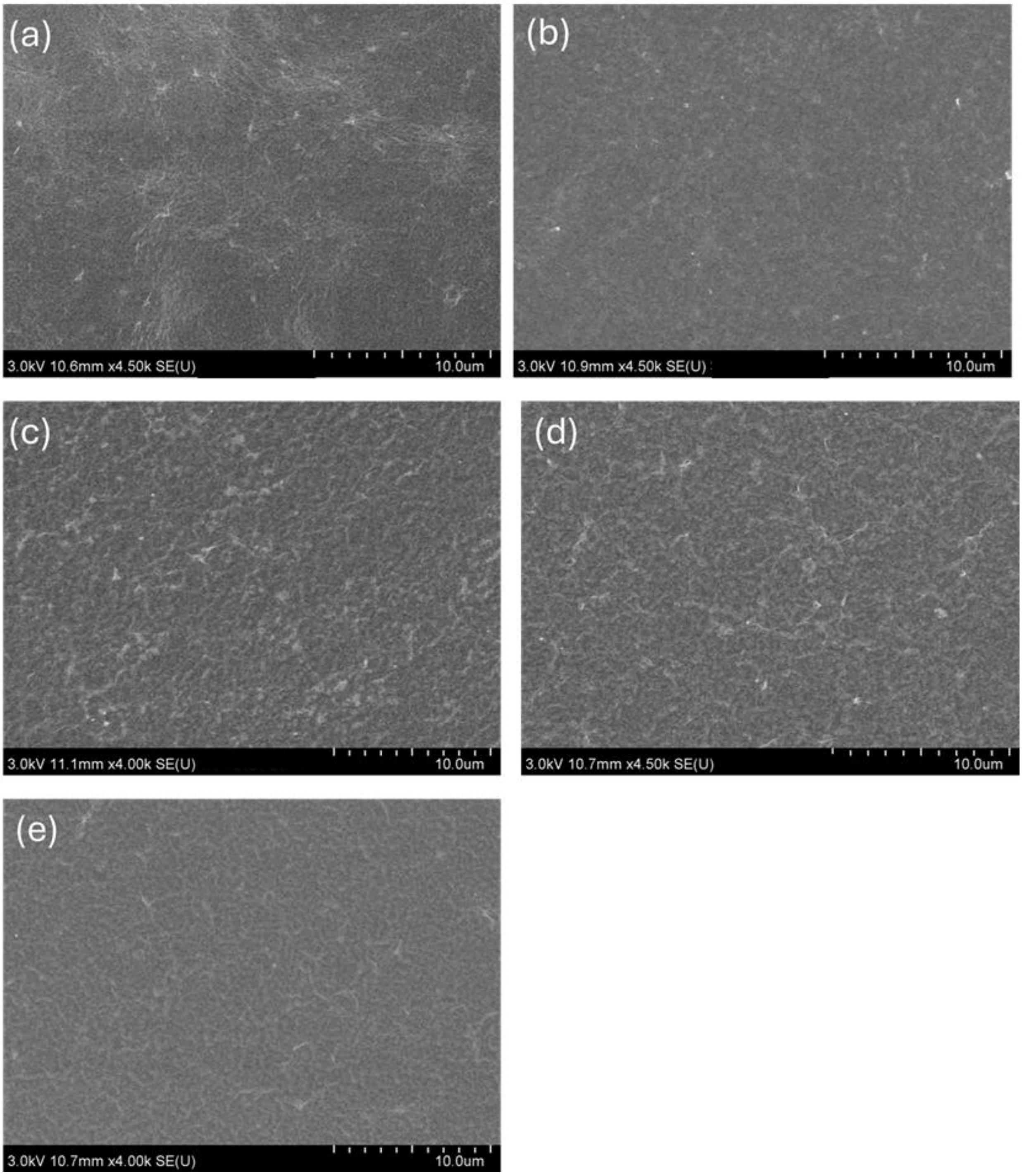

Figure S13. Top view SEM images of (a) rGO, (c) rGO-TZ1, (c) rGO-TM1, (d) rGO-AZ1, and (d) rGO-AZ2 membranes.

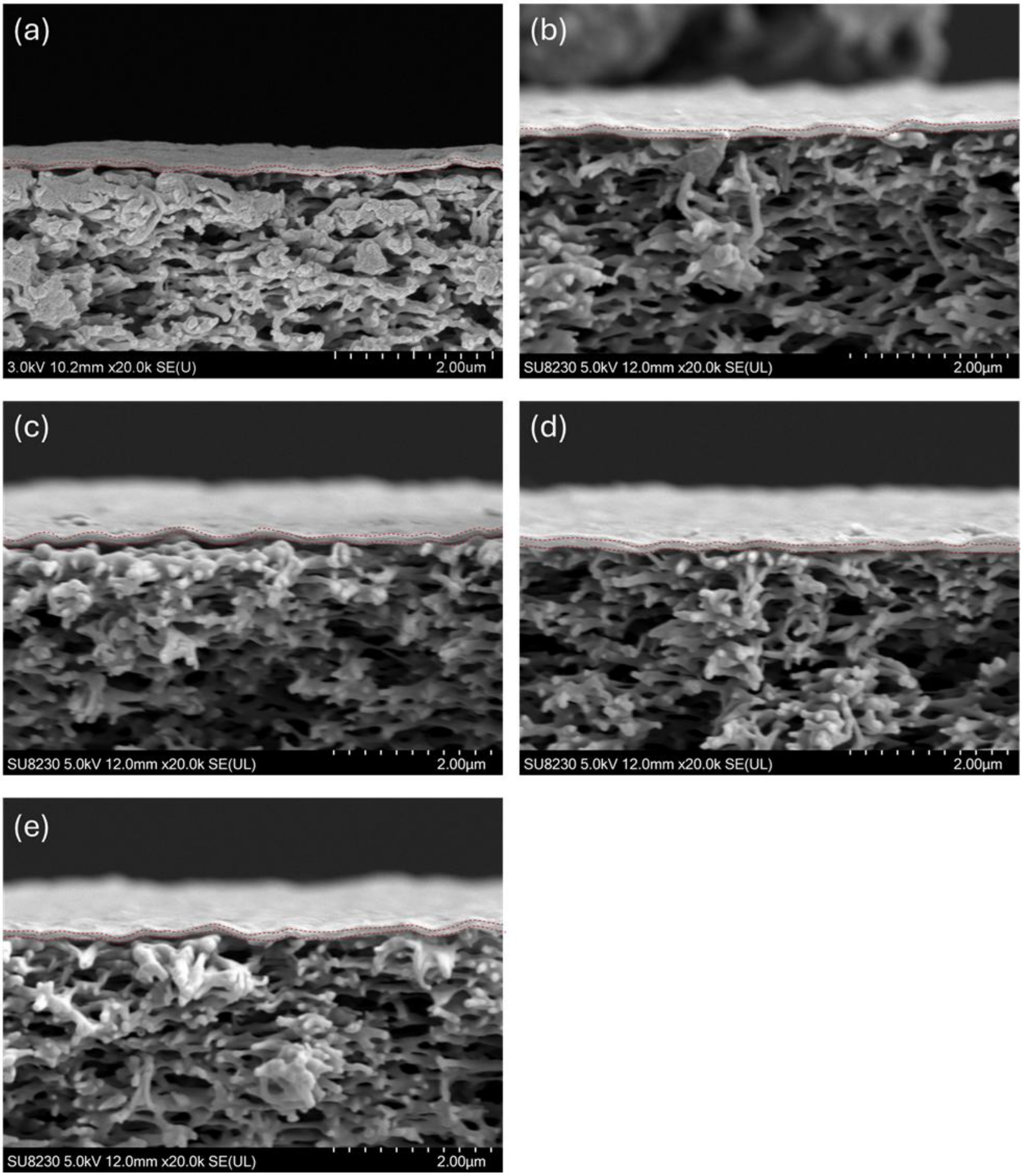

Figure S14. Cross-section SEM images of (a) rGO, (c) rGO-TZ1, (c) rGO-TM1, (d) rGO-AZ1, and (d) rGO-AZ2 membranes.

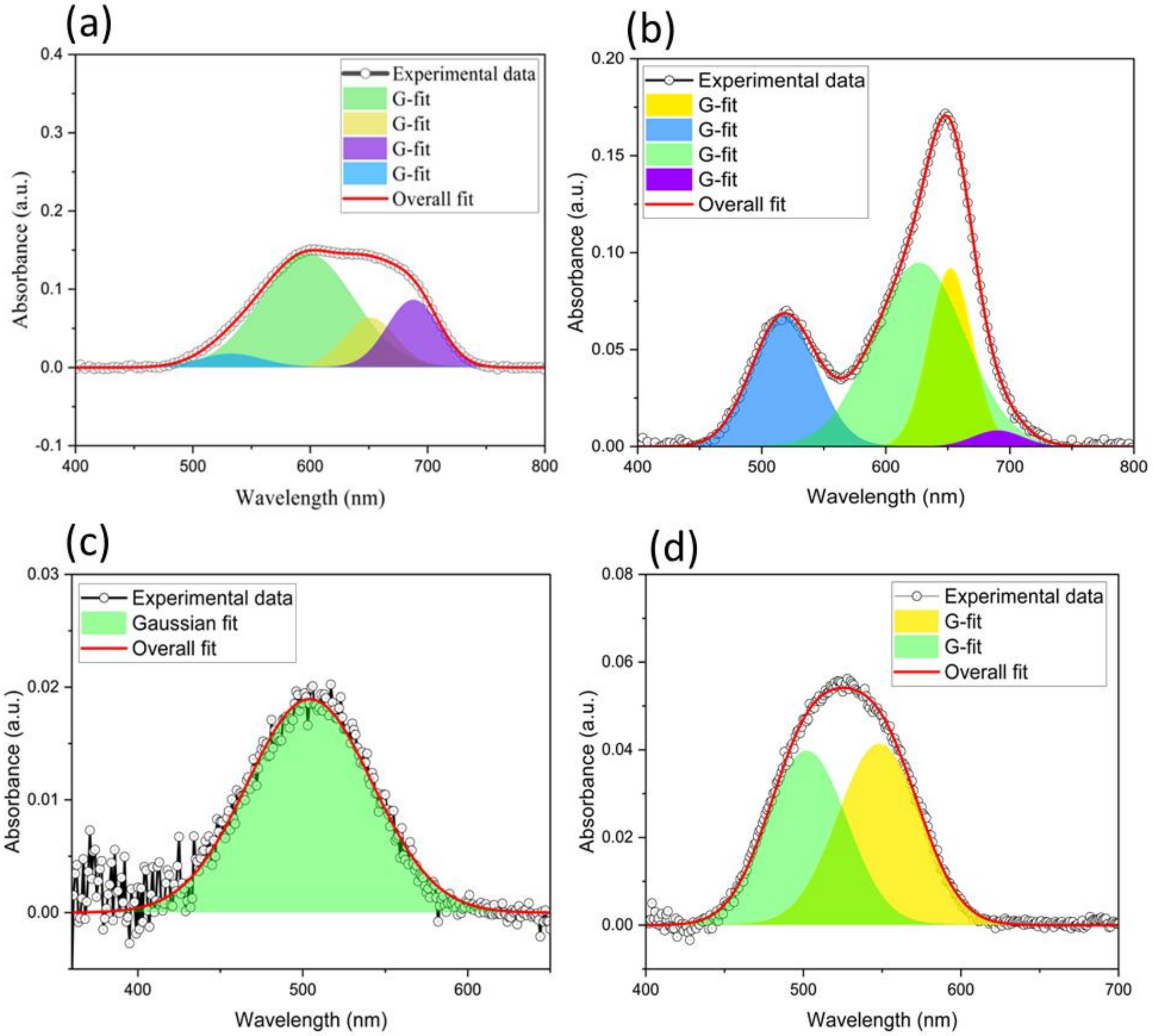

Figure S15. Peak fitted solid-state UV-Vis absorbance spectra of intercalated rGO-X membranes: (a) rGO-TZ1, (b) rGO-TM1, (c) rGO-AZ1, and (d) rGO-AZ2.

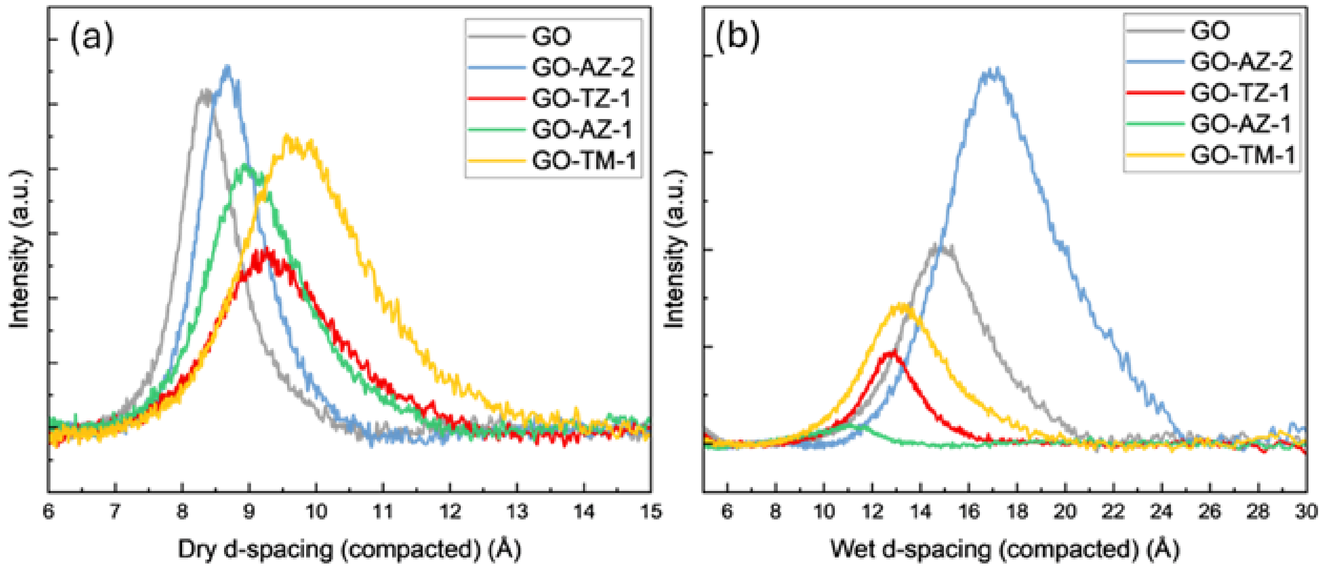

Figure S16. Raw XRD patterns of the GO-X membranes for (a) dry and (b) wet compacted situations.

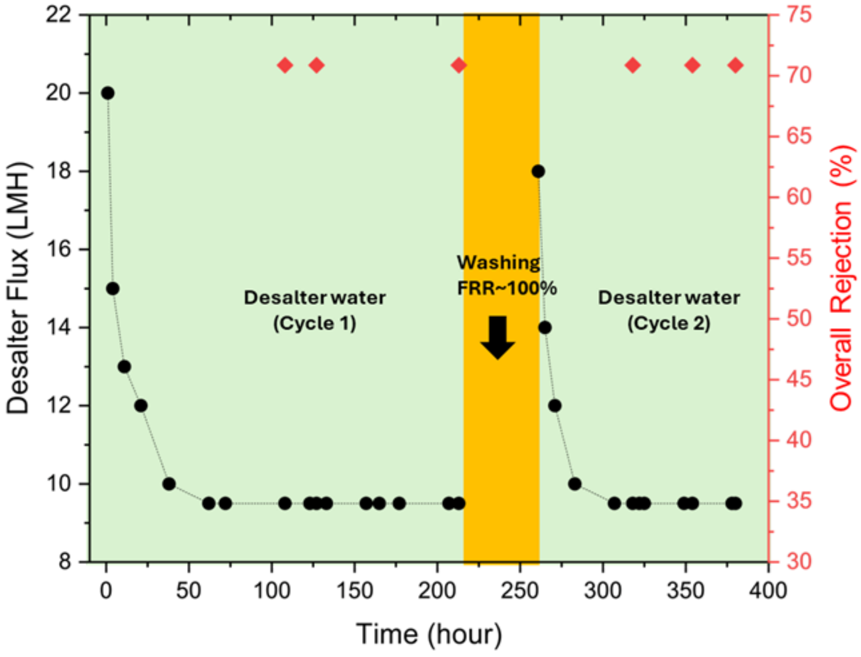

Figure S17. Long-term permeation performance of rGO-TM1 membrane during two cycles of desalter wastewater treatment with intermediate cleaning.

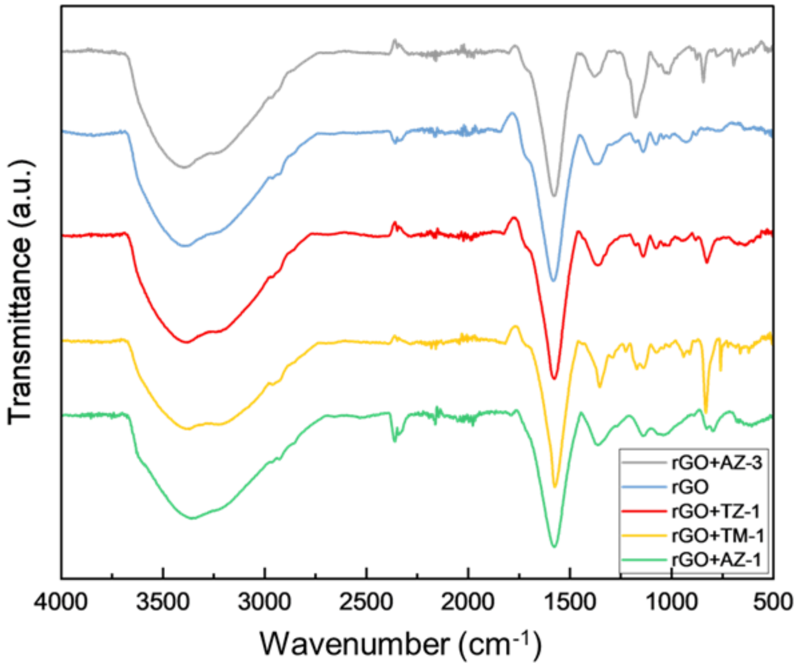

Figure S18. ATR-FTIR spectra of rGO and rGO-X free-standing films over the full range (4000–500 cm^{-1}).

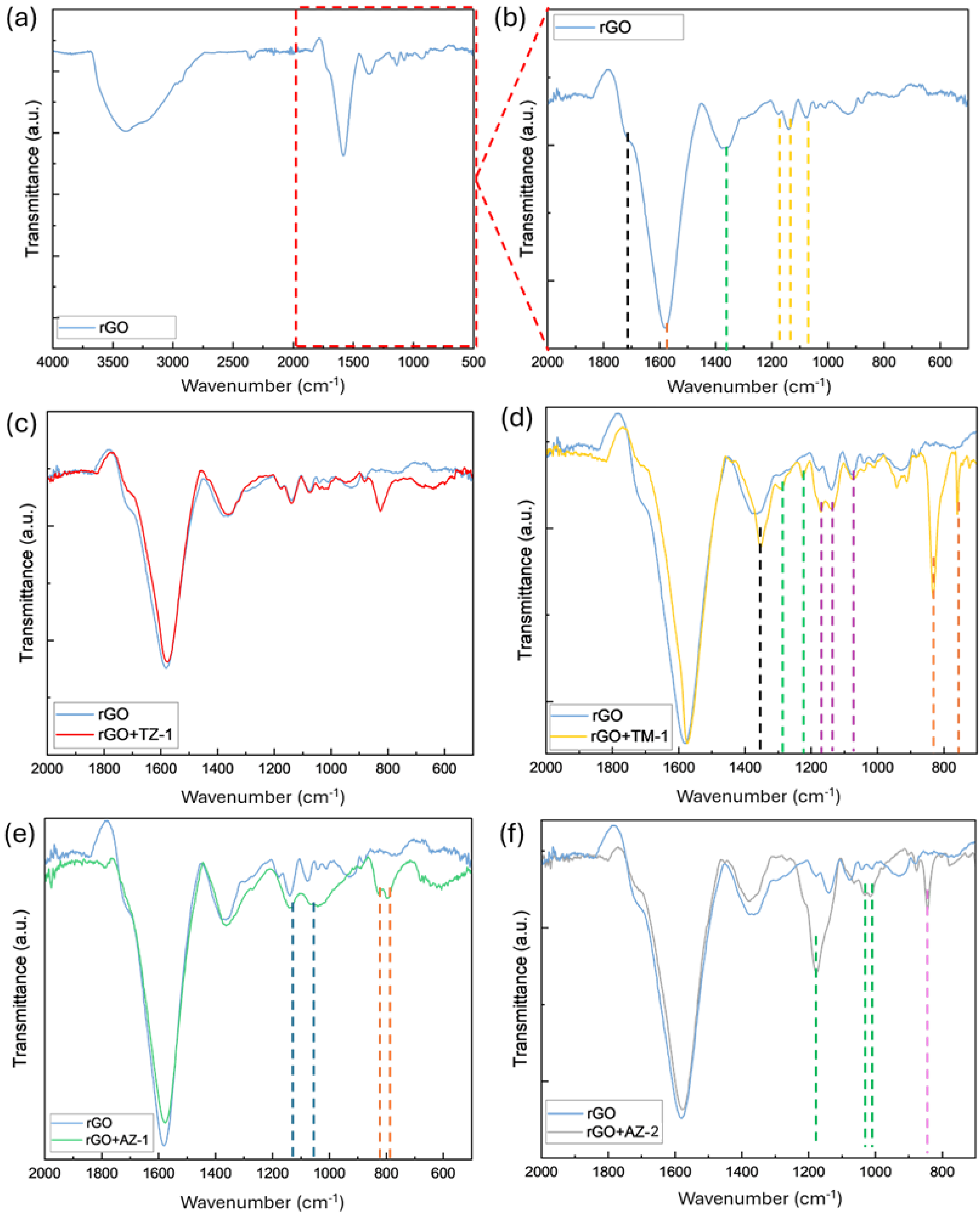

Figure S19. Detailed FTIR spectra of free-standing rGO films: (a-b) rGO, (c) rGO-TZ1, (d) rGO-TM1, (e) rGO-AZ1, and (f) rGO-AZ2.

3. Supporting Tables

Table S1. Molecules used in molecular weight cut-off measurements.

Molecules	Molecular Weight (Da)	Acidic/Basic
Xylose	150	Neutral
Glucose	180	Neutral
Toluidine Blue O	306	Cationic
Allura Red AC	496	Acidic
Methyl Blue	800	Acidic

Table S2. Numerical data corresponding to **Figure 4a**. Na_2SO_4 and NaCl rejection and flux data from intercalated rGO membranes in this work, and literature works in the 2021-2025 period. Data from earlier works shown in Figure 4a are available in Refs. 26,42,50,65–119. All the reported measurements were carried out at ambient temperature.

Membrane	Na_2SO_4 Feed (M)	Na_2SO_4 rejection (%)	NaCl feed (M)	NaCl rejection (%)	Flux* ($Lm^{-2}h^{-1}$)	Pressure (bar)	Reference
rGO (baseline of this work)	0.01	84	0.01	75	31	50	This work
	0.05	81	0.05	70	29	50	This work
	0.1	79	0.1	68	29	50	This work
	0.5	58	0.5	46	22	50	This work
rGO+TM-1	0.01	97	0.01	89	29	50	This work
	0.05	97	0.05	81	26	50	This work
	0.1	97	0.1	79	26	50	This work
	0.5	91	0.5	62	12	50	This work
rGO+AZ-1	0.01	97	0.01	86	28	50	This work
	0.05	97	0.05	81	26	50	This work
	0.1	97	0.1	79	25	50	This work
	0.5	91	0.5	60	15	50	This work
rGO+AZ-2	0.01	81	0.01	68	86	50	This work
	0.05	78	0.05	60	74	50	This work

	0.1	77	0.1	50	70	50	This work
	0.5	53	0.5	25	64	50	This work
TPPH/GO-1.0	0.0141	94	0.034	49	4.2	10	[26]
TPPA/GO-1.0		96		40	4		
TPP/GO-1.0		87		36	11.5		
TPPC/GO-1.0		94		45	7.5		
TPPF/GO-1.0		91		35	10.5		
TPPS/GO-1.0		89		34	12		
rGO-40	0.01	70	-	-	55	50	[42]
rGO-70		79			38		
rGO-100		84			30		
rGO-130		88			22		
GO	0.01	84	0.01	71	48	50	[50]
	0.1	72	0.1	52	29	50	[50]
	0.5	51	0.5	33	20	50	[50]
GO+9wt% TZ-1	0.01	90	0.01	81	21	50	[50]
	0.1	87	0.1	70	15	50	[50]
	0.5	81	0.5	48	8	50	[50]
PI/G_{500}	-	-	0.051	95	15.14	2.41	[65]
	-	-	0.103	82.11	9.88	2.41	
	-	-	0.154	76.82	7.44	2.41	
	-	-	0.205	71.01	6.11	2.41	
G1c10T60			0.00855	90	1	10	[66]
m-V-120-GOM	-	-	0.00855	95	0.5	0.2	[67]
	-	-	0.0171	90			
	-	-	0.0342	80			
	-	-	0.0856	65			
ArGO-PSSNa	-	-	0.005	95	112.5	5	[68]
ArGO	-	-	0.005	22	45	5	
rGO	-	-	0.005	18	12.5	5	
GO	-	-	0.005	29	25	5	
GO_0.1	0.01	46.6	0.01	26.7	-	5	[69]
GO-mimG_0.5	0.01	92.1	0.01	46.2	13	5	
GO/g-C_3N_4-3	0.02	92.5	0.02	87	152	5	[70]

GO 100 FLG	-	-	0.034	85	17	50	[71]
GO 35 FLG	-	-	0.0034	76	-	50	
	-	-	0.034	83	22	50	
	-	-	0.17	57	-	50	
	-	-	0.51	35	-	50	
GO 25 FLG	0.014	87	0.034	79	34	50	
	-	-	0.034	79	12	20	
rGO	0.0085	97	0.0085	89	2.2	10	[72]
GQD/rGO	0.0085	95	0.0085	80	4.9	10	
GQD-Ag/rGO	0.0085	95	0.0085	76	11.2	10	
Ag/rGO	0.0085	85	0.0085	50	13.6	10	
c-GO/PAN	0.04	51	-	-	30	8	[73]
rGO-10	0.014	98	-	-	-	56	[74]
	0.063	90	-	-	-	36	
GO: phenolic nanomesh (1:1)	0.0035	97	0.0085	40	165.6	1	[75]
$(PEI/GO)_3$	0.007	68	0.017	27	-	6	[76]
GO@PAMAM@Ag (TFN-0.02)	0.007	90	-	-	45	8	[77]
rhGO	0.01	69	-	-	210	10<br>50	[78]
rsGO-70	0.01	72	-	-	155	50	[79]
rmGO-70	0.01	79	-	-	38	50	
rmGO-130	0.01	87	-	-	18	50	
rlGO-130	0.01	82	-	-	9	50	
rsGO-130	0.01	87	-	-	82	50	
	0.05	73	-	-			
	0.1	66	-	-			
GO	-	-	0.01	55	20.8	0.8	[80]
AA/GO	-	-	0.01	87.5	268	0.8	
GO<br>GO-COF1	0.0141	39.2<br>10.2	0.0342	11.3<br>2.9	1<br>195	1	[81]
TFC	-	-	0.0171	94.4	6.1	5	[82]
TFN-$GO_{0.5}$	-	-	0.0171	93.8	8.15	5	
TFN-$GOP_{0.25}$	-	-	0.0171	95.1	9.85	5	
TFN-$GOP_{0.5}$	-	-	0.0171	96.3	12.65	5	
TFN-GOP_1	-	-	0.0171	91.03	12.35	5	

GS-Sr-3.0	0.014	1.01	0.034	0.75	200	5	[83]
GO/HTGO-0.7	0.00704	26.7	0.0171	15.11	100	1	[84]
DTGO	0.025	18	0.025	6	60	5	[85]
PAL_1/GO	0.0141	89.4	0.0352	78.1	4.8	1.25	[86]
PAL_2/GO	-	-		74.3			
PAL_3/GO	-	-		72.8			
PVP	0.00034	38	-	-	11	7	[87]
PVP/GO-0.1		52	-	-	40		
PVP/GO-0.2		60	-	-	53		
PVP/GO-0.3		72	-	-	57		
PVP/GO-0.4		90	-	-	72		
PVP/GO-0.5		59	-	-	62		
GO-HEMA	-	-	0.034	82	112-120	4.8	[88]
iTC02	0.0070	98	0.0171	23	106.5	5	[89]
NH_2-h-BN	-	-	0.0171	2	300	2	[90]
GOQD/NH_2-h-BN	-	-		3	280		
GO/NH_2-h-BN	-	-		5	170		
GO	-	12.4	-	-	50	1	[91]
GO-DDA	-	1.1	-	-	20	1	
M-0	-	-	0.0171	20	40	8	[92]
M-1	-	-		27	72	8	
M-2	-	-		30	88	8	
M-3	-	-		35	96	8	
M-4	-	-		48	98	8	
M-0	-	-		15	27	3	
M-1	-	-		28	60	3	
M-2	-	-		41	75	3	
M-3	-	-		42	105	3	
M-4	-	-		42	132	3	
Pristine GO	-	79	0.01	37	3	6	[93]
H_2O_2 3h	-	77		35	10.8		
H_2O_2 6h	-	75		32	29.4		
H_2O_2 9h	-	72		30	96		
rGO/CON-0	0.00704	48	-	-	-	1	[94]
rGO/CON-30		38	-	-	-		

rGO/CON-50		38	-	-	-		
rGO/CON-70		25	-	-	-		
rGO/CON-100		10	-	-	-		
dHGO200/PDA2-15	0.0141	12	-	-	136	1.7	[95]
dHGO200/PDA2-15	**0.211**	3	-	-	136		
TFC	-	-	0.0171	30.2	38	6	[96]
TFC-GO	-	-		45.6	58		
TFC-ZGO$_{0.7}$	-	-		54.4	70		
TFC-ZGO$_{0.9}$	-	-		57.4	88		
EB@GO/PES	0.000704	95.24	-	-	58.2	5	[97]
GO_0.5	0.0141	22.5	-	-	2.1	40	[98]
GO_1.0		25	-	-	2.5	40	
GO_2.0		25	-	-	3	40	
rGO-FG-0	0.01	42	0.01	40	16.2	2	[99]
	0.03	30	0.03	35			
	**0.1**	25	**0.1**	19			
rGO-FG-4.69	0.01	90	0.01	84	15.9		
	0.03	75	0.03	72.5			
	**0.1**	65	**0.1**	52			
rGO-FG-9.27	0.01	90	0.01	80	19.8		
	0.03	78	0.03	71			
	**0.1**	64	**0.1**	51			
rGO-FG-15.67	0.01	88	0.01	80	30.3		
	0.03	75	0.03	70			
	**0.1**	60	**0.1**	50			
rGO-FG-19.85	0.01	82	0.01	79	38.6		
	0.03	73	0.03	70			
	**0.1**	60	**0.1**	48			
rGO-FG-21.34	0.01	81	0.01	80	36.8		
	0.03	70	0.03	70			
	**0.1**	60	**0.1**	50			
NGO-0.54	0.014	49.8	0.034	17.5	16.9	10	[100]
NGO-0.41		70.9		41.5	8.6		
NGO-0.32		87.7		56.6	6.5		
NGO-0.25		89		60.1	2.8		
NGO-0.20		90.4		70.5	1.3		
NPGO-0.10		65.1		17	15.6		
NPGO-0.14		70.2		14.1	11.3		
NPGO-0.16		69.7		14.9	10.4		
NPGO-0.18		67.1		11.3	12.5		
NPGO-0.15		72.7		19.1	13.2		

NPGO-0.17		65.2		16.6	17.2		
NPGO-0.19		73.5		13	16.5		
NPGO-0.20		72.4		16.8	15.9		
PPD@GO-1:0	0.0070	81	0.017	9	4	4	[101]
PPD@GO-1:1		82		9.5	11.2		
PPD@GO-1:3		83		10.5	12.4		
PPD@GO-1:6		84		10	13.6		
PPD@GO-1:9		83.5		10	12.8		
PPD@GO-1:12		83.5		10	12.6		
rGO	0.0070	85	0.0171	30	0.6	4	[102]
c-GO/CON		98		82	1.5		
L-GO	0.05	64	0.05	39	-	10	[103]
S-GO		81		55	-		
S-rGO		90		59	-		
Pristine GO	0.00704	52	0.017	14	12	2	[104]
PG-100		28.75		8.05	94		
GO-WKF	0.000704	7.2	0.00171	7	31-42	8	[105]
TFC	0.0141	91.57	0.0342	88.2	11.3	7.5	[106]
TFGO50		90.6		86.8	21.2		
TFβGO50		93.06		91	22.65		
SAGO@PVDF	0.00704	48	0.017	27	450	2.07	[107]
Glu2/GO	0.0141	96.42	-	-	6.9	10	[108]
GOLB	0.001	5	0.001	10	1010-1165	1	[109]
pGO		23		43	10-23		
m-IPN	-	-	0.034	96.8-98.9	225-275	7	[110]
icGO-K	0.00704	70.7	0.0171	81.8	7	2	[111]
GO_{pH3}	0.00704	89	0.0171	45	9.75	6.5	[112]
GO_{pH7}		84		44	10.4		
GO_{pH11}		81		41	7.8		
GP_{pH3}		18		50	16.25		
GP_{pH7}		12		45	13.65		
GP_{pH11}		11		44	33.15		
GO/icONs (M1)	0.0070	24	0.0171	12	75	1	[113]

PA-GO(0.1)/PES	0.00704	78	0.0171	69	14	4	[114]
GO-PAMAM	0.0141	98.4	0.034	52	11.26	5-8	[115]
GO_1-$MPD_{0.5}$	0.00141	35.5	0.00342	28	17.2	4	[116]
GO/GO_1-$MPD_{0.5}$-0.25		38		37.5	14.6		
Arg@GO	-	-	0.00025	99.5	145	1	[117]
L-GO	0.00704	40	0.0171	28	12-17	1	[118]
L-rGO		88		52	4-6		
S-rGO		87		48	14-27		
rGO	-	-	0.0171	75	10.2	6	[119]
GQDs-rGO	-	-		66	19.2		
GQDs/rGO	-	-		89	8.4		

*All the fluxes are rounded to the nearest whole number.

Table S3. Chemical analysis of the petroleum desalter wastewater (A) feed stream used in **Figure 4**, and the permeate obtained from varying thickness rGO-TM1 membranes.

Overall Chemical Analysis and Inorganic Anion Analysis	Feed Concentration (g/L)	Permeate Concentration (g/L) rGO-TM1-100%	Permeate Concentration (g/L) rGO-TM1-50%	Permeate Concentration (g/L) rGO-TM1-25%
Total organic carbon (Coulometric titration)	0.028	0	0	0
Total inorganics	2.87	0.30	0.45	0.51
Total suspended solids	0.065	0	0	0
Total solids	2.96	0.3	0.45	0.51
**Salts (Ion Chromatography)**				
NaCl	0.38 (0.0065M)	0	0.0076	0.023
Na_2SO_4	1.05 (0.0074M)	0.011	0.021	0.032
Na_2CO_3	1.44 (0.0135M)	0.29	0.42	0.45

Table S4. Chemical analysis of the petroleum desalter wastewater (B) feed stream used in the long-term study (**Figure S17**), and the corresponding steady state permeate obtained from the rGO-TM1 membrane.

Overall Chemical Analysis and Inorganic Anion Analysis	Feed Concentration (g/L)	Permeate Concentration (g/L) rGO-TM1-100%
Total inorganics	5.82	1.64
Total suspended solids	0.2	0
Total solids	6.02	1.64
**Salts (Ion Chromatography)**		
NaCl	4.68 (0.08M)	1.40
Na_2SO_4	0.98 (0.0068M)	0.24
Na_2CO_3	0.16 (0.0015M)	0.0078

Table S5. Assignments of vibrational bands observed in the FTIR spectra of rGO and intercalated rGO membranes.

Wavenumber (cm-1)	Compound class		rGO	rGO+TZ1	rGO+AZ1	rGO+AZ2	rGO+TM1
3200-3550	O-H stretching (hydrogen-bonded)	Alcohol	✓	✓	✓	✓	✓
2920-2850	C-H stretching	Alkane	✓	✓	✓	✓	✓
1720	C=O stretching	Aldehyde<br>α,β-unsaturated ester<br>Aliphatic ketone<br>Carboxylic acid	✓	✓	✓	✓	✓
1585	C=C stretching	Cyclic alkene	✓	✓	✓	✓	✓
1350-1380	C-H bending<br>O-H bending	Aldehyde, Alkane<br>Alcohol, phenol,	✓	✓	✓	✓	✓
1285	C-N Stretching<br>C-O stretching	Aromatic amine<br>Aromatic ester					✓
1225	C-N stretching<br>C-O stretching	Amine<br>Vinyl ether					✓
1170	C-O stretching	Ester, tert-alcohol				✓	✓
1140	C-O stretching	tert-alcohol			✓		✓
1130, 1180, 1070	C-O stretching	Alcohol, ester	✓	✓	✓		
1080-1016	C-O stretching	prim-alcohol			✓	✓	✓
845<br>875<br>890	C=C bending	Alkene				✓	
830	C-Cl stretching	Halo compound		✓	✓		✓
791	C-H bending	1,2,3-trisubstituted			✓		
760	C-Cl stretching<br>C-H bending	Halo compound<br>1,2,3-trisubstituted					✓

4. Author Contributions

This work was conceived by S. N. Materials synthesis, membrane fabrication, membrane permeation, and materials/membrane characterization were performed by M. S. The manuscript writing was led by M. S., and both authors contributed to the manuscript editing.
